# Nanoencapsulation of nutraceuticals: enhancing stability and bioavailability in functional foods

**DOI:** 10.3389/fnut.2025.1746176

**Published:** 2026-01-12

**Authors:** Minglu Hao, Xiaonan Tan, Kang Liu, Ning Xin

**Affiliations:** Qingdao Hengxing University of Science and Technology, Qingdao, China

**Keywords:** bioavailability, food nanotechnology, functional foods, nanocarriers, nanoencapsulation, nutraceuticals

## Abstract

While nutraceuticals hold great promise for improving health, their efficacy is often limited by the poor stability and low bioavailability of many bioactive compounds. Nanoencapsulation has emerged as a transformative solution to these challenges, involving nanoscale carriers that protect sensitive nutrients from degradation and enhance their absorption. This review provides a comprehensive overview of nanoencapsulation strategies in the food and nutrition domain. We outline the historical development of key nanocarrier systems, from early liposomal delivery vehicles to advanced lipid nanoparticles, nanoemulsions, and biopolymer-based nanoparticles engineered for improved nutraceutical delivery. The physicochemical and biological mechanisms responsible for enhancing the performance of nanoencapsulation systems are systematically elucidated. Nanoencapsulation can shield bioactives from light, heat, oxidation, and pH extremes. It also improves oral bioavailability through enhanced solubility, controlled release, mucoadhesion, endocytosis-mediated uptake, and lymphatic transport that bypasses first-pass metabolism. Key examples of nanoencapsulated nutraceuticals in functional beverages, dairy products, dietary supplements, and other foods are highlighted, illustrating the growing commercial applications of food nanotechnology. We also discuss challenges related to manufacturing scale-up, safety and toxicology, regulatory oversight, and consumer acceptance of nano-enabled nutraceuticals, and we present emerging trends such as co-encapsulation of synergistic ingredients and precision nutrition approaches. By integrating advances in nanotechnology with nutritional science, nanoencapsulation is poised to significantly amplify the health benefits of functional foods and nutraceutical products in the coming years, paving the way for more effective functional diets and improved public health.

## 1 Introduction

With the increasing global awareness of health and the continuous improvement in living standards, modern consumers are no longer satisfied with the basic nutritional value of food. Instead, there is a growing demand for functional foods and nutraceuticals-products that not only provide essential nutrients but also contribute to the prevention of diseases and the promotion of health. This paradigm shift in consumer preference has catalyzed a profound integration of nutritional science and food technology, thereby accelerating innovation across both disciplines.

However, the translational gap between the *in vitro* efficacy and the *in vivo* performance of many bioactive compounds (BACs) remains a major bottleneck in the development of efficacious nutraceutical products (1, 2). Numerous BACs derived from natural sources- such as curcumin, resveratrol, omega-3 polyunsaturated fatty acids, various vitamins, and polyphenolic compounds- have been extensively demonstrated to exert potent antioxidant, anti-inflammatory, and other biofunctional activities in controlled experimental settings (3–8). Nevertheless, these effects are often markedly attenuated following oral administration due to their poor stability and limited bioavailability under physiological conditions.

The core of this issue lies in the inherent physicochemical limitations of many BACs. A substantial proportion of these compounds are lipophilic in nature and exhibit extremely low solubility in the aqueous environment of the gastrointestinal tract. Furthermore, they are chemically unstable and highly sensitive to external factors such as light, heat, oxygen, and pH fluctuations, leading to substantial degradation during processing, storage, and gastrointestinal transit (9–15). Even if a fraction of the active ingredient reaches the small intestine intact, its absorption is often severely restricted due to unfavorable molecular size, poor intestinal permeability, and extensive hepatic first-pass metabolism. Collectively, these factors result in exceedingly low bioavailability, defined as the fraction of the ingested compound that reaches systemic circulation in an active form, thereby limiting the therapeutic and nutritional efficacy of such agents.

In response to these challenges, nanoencapsulation has emerged as a promising and transformative strategy. This technology involves the encapsulation of active compounds within nanocarriers- typically ranging from 1 to 100 nanometers- constructed from food- grade biocompatible materials. These nanocarriers function as protective matrices, isolating sensitive BACs from deleterious external conditions, enhancing their dispersion in aqueous media, and improving their physicochemical stability (16, 17). More importantly, the nanoscale dimension and high surface-area-to-volume ratio of these carriers facilitate enhanced intestinal uptake via multiple mechanisms, and in some cases, may even enable partial circumvention of hepatic first-pass metabolism (18, 19). These advantages have been shown to dramatically improve the oral bioavailability of encapsulated compounds, thereby restoring and amplifying their physiological effects *in vivo*.

Several reviews have appeared in recent years on nanoencapsulation of food nutraceuticals, but each has addressed specific subsets of this broad topic. For instance, prior articles have focused on immune-boosting encapsulated foods in the context of COVID-19 or on advances in liposome formulation techniques and safety aspects (20–22). By contrast, the present review addresses a notable gap: no comprehensive work has yet integrated the latest innovations in nanoencapsulation specifically to highlight their dual impact on both stability and bioavailability of nutraceuticals across various carrier systems and functional foods. Building upon foundational studies in nutraceutical delivery nanotechnology, we incorporate new insights from the past several years – including emerging nanocarriers (lipid- and biopolymer-based), co-encapsulation of synergistic ingredients, and real-world considerations such as scale-up and regulatory status – that have not been collectively examined in previous reviews. In doing so, we aim to provide a timely and holistic perspective that will benefit researchers and industry practitioners alike. We also describe the global research and development trend in nanoencapsulation for functional foods. Over the last decade, scientific and commercial interest in this area has grown exponentially. Nanotechnology-based encapsulation is now recognized as a cutting-edge approach for improving the shelf-life, stability, and bioavailability of bioactive compounds in foods. This surge in activity – reflected by the increasing number of publications, patents, and nano-enabled functional products – underscores the importance of the present review in charting current progress and future directions in nutraceutical nanoencapsulation.

## 2 Historical trajectory of nanoencapsulation: key developments and breakthroughs

### 2.1 Conceptual emergence and theoretical foundations

The intellectual origin of nanoencapsulation can be traced back to the mid-20th century. In 1959, Nobel Laureate in Physics Richard Feynman delivered his seminal lecture titled *“There’s Plenty of Room at the Bottom,”* in which he proposed the revolutionary idea of manipulating matter at the molecular and atomic scales (23). Although his visionary concept was initially directed toward applications in physics and electronics, it laid the philosophical and scientific groundwork for all subsequent advances in nanotechnology.

Concurrently, in the domain of health and nutrition, a paradigm shift was unfolding regarding the relationship between dietary components and therapeutic outcomes. In 1989, Dr. Stephen De Felice, founder of the Foundation for Innovation in Medicine, introduced the term “nutraceutical”- a portmanteau of “nutrition” and “pharmaceutical.” He defined nutraceuticals as “a food or part of a food that provides medical or health benefits, including the prevention and/or treatment of disease” (24). This definition elevated the functional potential of foods to a quasi-pharmaceutical level and signaled a new era of health-oriented food innovation.

The convergence of nanotechnology’s core principle of microscale precision and the nutraceutical concept of targeted health benefits provided a fertile theoretical framework for the development of nanoencapsulation in the context of food and nutrition. It was this intersection- between the ability to manipulate matter at the nanoscale and the aspiration to deliver precise, bioactive functionalities through food- that catalyzed the emergence of nanoencapsulation as a transformative strategy in functional food science.

### 2.2 Key technological milestones in nanoencapsulation for nutraceuticals

**Figure 1:**
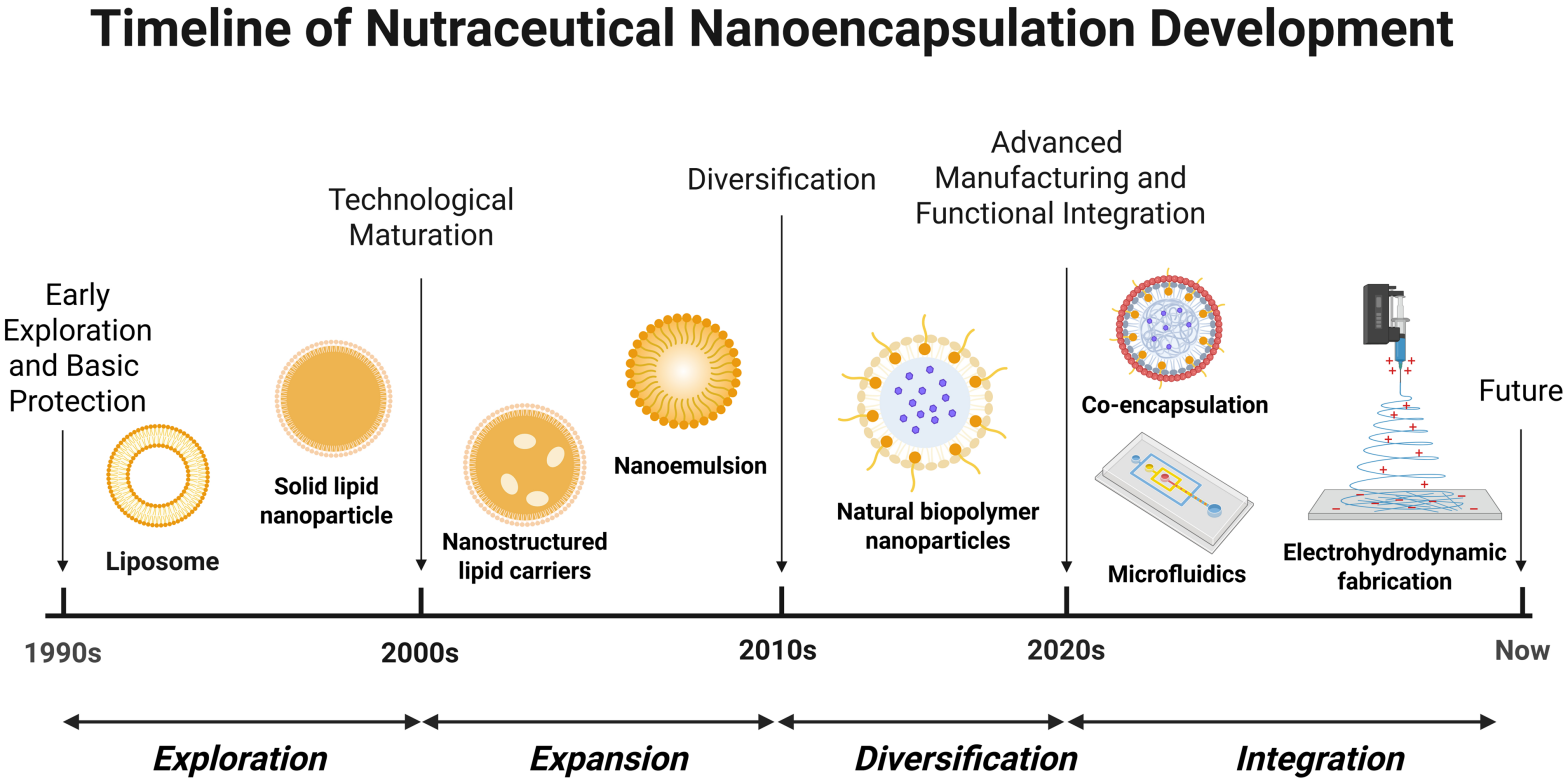
The timeline of representative technologies in nutraceutical nanoencapsulation. Created with BioRender.com.

The development of nanoencapsulation technologies in nutraceuticals has progressed through several key phases, reflecting a transition from basic protection of ingredients to advanced, multifunctional delivery systems. What began as a concept borrowed from pharmaceutical drug delivery in the 1990s has evolved into sophisticated, targeted nano-delivery platforms today. Below we expand on each phase of this technological evolution, outlining how nanoencapsulation moved from initial explorations to practical implementation in nutraceutical products. The timeline of representative technologies in nutraceutical nanoencapsulation is shown in Figure 1.

#### 2.2.1 Early exploration and basic protection (1990s)

In the 1990s, nutraceutical scientists first explored nanocarriers largely inspired by pharmaceutical advances. Liposomes were among the earliest nanoencapsulation vehicles adopted in food science. These spherical vesicles made of phospholipid bilayers could encapsulate both water-soluble and fat-soluble nutraceutical compounds, thereby protecting sensitive ingredients from degradation (25–27). Hydrophilic nutraceuticals (e.g., vitamins like vitamin C or B-complex) can be trapped in the inner water phase, while lipophilic molecules (e.g., omega-3 oils, curcumin) embed within the hydrophobic lipid bilayer. This dual encapsulation capability is well documented. For instance, a recent review in Foods notes that “liposomes can encapsulate both hydrophilic and lipophilic compounds” owing to their concentric phospholipid bilayer structure (28). Similarly, Subramani and Ganapathyswamy highlight that lipid-based carriers like liposomes are a “superior choice for encapsulation of sensitive ingredients” of varied polarity- covering hydrophobic and hydrophilic bioactive molecules alike (29). Liposomes’ biocompatible, membrane-like structure offered a promising strategy for shielding labile nutrients (like vitamins or polyphenols) from light, oxygen, and stomach acidity during delivery. For example, vitamin C is highly prone to oxidation and loses potency quickly in fortified foods. Studies have shown that liposomal entrapment of vitamin C dramatically extends its stability- increasing shelf-life from just a few days to up to 2 months by protecting it from pro-oxidant factors like metal ions and enzymes (30). Another study of Gopi and Balakrishnan (31) indicated that the oral delivery of vitamin C encapsulated in liposomes registered 1.77 times more bioavailable than the non-liposomal vitamin C. Likewise, antimicrobials and enzymes entrapped in liposomes retain activity longer in food matrices. Benech et al. (32) reported that liposome-encapsulated nisin (a natural preservative) had improved stability and efficacy against Listeria in cheese compared to free nisin. Early on, however, translating liposome technology into commercial nutraceutical products proved challenging; it wasn’t until the late 1990s that liposomal supplements and functional foods began achieving market success (33). This delay was due in part to stability issues and manufacturing hurdles that had to be overcome.

Soon after liposomes, researchers developed solid lipid nanoparticles (SLNs) as a second innovation in nutraceutical nanoencapsulation. Conceived by blending concepts from emulsions, liposomes, and polymeric nanoparticles, SLNs use lipids that remain solid at room temperature to form a nanocarrier matrix (34, 35). The solid lipid core of an SLN provides a more rigid, protective environment for encapsulated nutrients, significantly improving their physical and oxidative stability compared to conventional emulsions or even liposomes (36–38). This stability meant, for example, that a fragile antioxidant or essential oil could be shielded against rancidity and volatilization more effectively in an SLN. In a study by Yu et al. (39), curcumin-loaded solid lipid nanoparticles (SLNs) formulated with medium- and long-chain diacylglycerol or tripalmitate showed over 87% encapsulation efficiency, improved stability under heat and varying pH conditions, and significantly greater *in vitro* bioaccessibility compared to unencapsulated curcumin. Another example is a study that reported “Curcumin Encapsulated Lipidic Nanoconstructs (CLEN)” achieving an approximately 1.4-million-fold increase in aqueous solubility and about a 70-fold improvement in oral bioavailability compared to free curcumin (40). Additionally, SLNs were found to enable a controlled-release of the active compounds; the solid matrix slows down diffusion, allowing nutrients to be released over a sustained period rather than all at once. An important advantage of SLNs is that they are made from physiological, biodegradable lipids (e.g., triglycerides and fatty acids), many of which are Generally Recognized as Safe (GRAS) for food use (41, 42). This biocompatibility minimizes toxicity concerns and was a key factor in their early adoption for nutraceutical applications. By the end of the 1990s, the introduction of SLNs marked a critical advance- researchers had moved beyond simple emulsions to true nanostructured carriers that offered both protection and better retention of sensitive nutraceutical ingredients.

In summary, these early studies collectively demonstrated that liposomal encapsulation could extend the shelf-life of labile nutrients and modestly enhance their bioavailability relative to non-encapsulated forms. These benefits arise from the phospholipid bilayer of liposomes acting as a protective barrier and absorption aid – the bilayer isolates the active compound from degradative factors (oxygen, low pH) and can fuse with biological membranes to facilitate uptake. Notably, the magnitude of bioavailability improvement varied across studies; a 1.77× increase for vitamin C (31), while significant, is less dramatic than enhancements reported with newer nanocarriers, indicating that formulation factors and the compound’s chemistry influence efficacy. Indeed, some early liposome formulations initially suffered stability issues (e.g., payload leakage, short shelf-life), and a few reports showed inconsistent results, highlighting minor contradictions in performance until manufacturing techniques were optimized. By comparing these studies, it became clear that careful optimization of liposomal formulations (e.g., using high-purity phospholipids, proper size control) was required- a fact underscored by the delayed commercial success of liposomal nutraceuticals until the late 1990s. Once these hurdles were addressed, later studies consistently confirmed improved nutrient stability and efficacy with liposomal delivery compared to free compounds, validating liposomes as a foundational nanocarrier for nutraceuticals.

#### 2.2.2 Technological maturation and diversification (early 2000s to present)

Entering the 2000s, nanoencapsulation technologies underwent rapid maturation and diversification, driven by growing industrial demand for more effective delivery of nutraceuticals. One milestone was the development of nanostructured lipid carriers (NLCs) in the early 2000s as a second-generation lipid nanocarrier. NLCs were designed specifically to address some limitations of the first-generation SLNs- such as limited loading capacity and the tendency of some active compounds to be expelled from the solid lipid matrix during storage. To solve this, NLCs incorporate a blend of solid *and* liquid lipids, creating an imperfect or non-uniform crystalline structure in the nanoparticle core (43, 44). This less orderly lipid arrangement leaves extra molecular “space” in the core, allowing higher payloads of bioactive compounds to be stably encapsulated (45). The imperfect crystallinity also reduces the risk of expulsion: as the mixture of lipids solidifies, it does not form a densely packed crystal lattice, so there are voids and amorphous regions where nutraceutical molecules remain trapped. The result is that NLCs can carry more nutraceutical payload and maintain their stability over time better than SLNs. One formulation co-encapsulating omega-3 fish oil and *α*-tocopherol showed long-term stability (>30 days) with encapsulation efficiency over 60% (46). This innovation of blending lipids thus maintained the safety and biocompatibility of lipid carriers while improving performance, and NLCs quickly gained prominence in oral supplement formulations and functional foods (43).

Around the same time, nanoemulsion technology advanced into the nutraceutical arena. Nanoemulsions are ultrafine oil-in-water emulsions with droplet sizes typically in the 20–200 nm range. Researchers developed both high-energy methods (e.g., high-pressure homogenization, microfluidization, ultrasonication) and low-energy methods (e.g., spontaneous emulsification, phase inversion techniques) to reliably produce these *nanoscale* emulsions. The appeal of nanoemulsions lies in their ability to solubilize lipophilic nutraceuticals (such as curcumin, carotenoids, or essential oils) in a water-like format. Thanks to the tiny droplet size, nanoemulsions can be nearly transparent and are far more stable against creaming or phase separation than conventional emulsions. For example, accelerated-curcumin release studies indicate nanoemulsions prepared via high-pressure homogenization significantly enhance curcumin dissolution in lipophilic food simulants (47). This meant that previously “oily” nutrients could be incorporated into clear beverages, gels, and other aqueous products without affecting appearance or texture. Walker et al. (48) highlighted in their review that nanoemulsions are colloidal dispersions that can be engineered to exhibit excellent kinetic stability and high optical clarity- features that are particularly valuable for applications in various food and beverage products. This observation supports the use of nanoemulsions in functional foods, such as sports drinks, where they can provide a clear appearance while preventing oil- water separation. In a related study on yogurt-based beverages, the incorporation of *ω*-3 nanoemulsions significantly enhanced bioavailability without causing turbidity or phase separation (49). Nanoemulsions also often show improved bioavailability of actives because the small droplets have a huge surface area for digestion, and the encapsulated nutraceuticals can be more readily absorbed. The first decade of the 2000s saw nanoemulsions become a versatile platform in nutraceutical formulation, expanding the possibilities for delivering poorly water-soluble vitamins and phytochemicals in functional foods (50).

In parallel with lipid-based carriers, natural biopolymer nanoparticles gained increasing attention as “green” nanoencapsulation solutions. Scientists explored using food-grade proteins and polysaccharides to create nanocarriers, capitalizing on their biocompatibility and regulatory acceptance. Examples include protein-based nanoparticles made from whey protein isolate (51, 52), zein (corn protein) (53, 54), or casein (55, 56), and polysaccharide-based systems using chitosan (57), alginate (58, 59), or pectin (60, 61). These biomaterials are inherently non-toxic, biodegradable, and often already approved as food additives, making them attractive for nutraceutical delivery (62). Beyond their safety, many of these biopolymers impart special functional traits to the nanocarriers:

*Mucoadhesiveness*: Biopolymers such as chitosan and its quaternized derivatives, carbopol and hyaluronic acid can adhere to mucosal surfaces in the gut (63), forming a viscoelastic layer on the intestinal lining and prolonging the residence time of the encapsulated nutraceuticals. A mucoadhesive nanoparticle will stick to the intestinal lining, prolonging the residence time of the encapsulated nutraceutical and potentially improving absorption. A study of Kim et al. (64) showed that quercetin-loaded nanoparticles formulated with chitosan and gum arabic (QCG-NPs) exhibit significantly enhanced mucoadhesive properties *in vitro*, attributed to electrostatic interactions with the mucin layer. This increased adhesion promotes greater uptake of quercetin by intestinal cells and enhances its antioxidant activity. Another study of Raghunath et al. (65) on oral insulin delivery, chitosan-coated solid lipid nanoparticles were found to prolong retention time at the intestinal mucosa. Further analysis using confocal microscopy revealed their ability to penetrate deeply into intestinal tissues, leading to improved insulin bioavailability (65). Additionally, Yen et al. (66) proved that the use of high-molecular-weight chitosan as a carrier for delivering alginate/chitosan-layered PLGA nanoparticles via the intestinal route has shown promising results. This delivery system was found to facilitate intestinal absorption and enhance the bioavailability of the active compounds, further emphasizing the beneficial role of chitosan-based coatings in oral drug delivery (66). These findings highlight the mucoadhesive nature of chitosan as a key factor in prolonging the residence time of nanoparticles at the absorption site, thereby facilitating improved drug permeation and absorption.

*pH-responsiveness*: Polymers like alginate or pH-sensitive gelatin can be engineered to respond to gastrointestinal pH changes. For example, an alginate-chitosan coacervate nanoparticle might remain intact in the acidic stomach but then swell or dissolve at the higher pH of the small intestine, thereby releasing the payload at the desired site. This kind of enteric-triggered release protects acid-labile compounds through the stomach and targets their release to intestinal absorption sites. For instance, Rizwan et al. (67) provided a comprehensive review of pH-sensitive hydrogels, focusing on material selection, structural characteristics, and the mechanisms by which these systems swell or dissolve in response to pH changes within the gastrointestinal tract to regulate drug release. The review emphasized that carriers designed using ionizable polymers, such as alginate and gelatin, can remain stable in the acidic gastric environment and release their payload in alkaline conditions- an effective strategy for enhancing drug bioavailability (67). Bachta et al. (68) highlighted in their review that pH-sensitive nanoparticles- commonly composed of materials such as alginate and chitosan- can modulate their size, solubility, and drug release rate in response to pH-dependent physicochemical changes. This adaptability significantly enhances drug stability within the gastrointestinal tract and promotes targeted absorption (68).

*Enzymatic or microbial stability:* Biopolymer-based carriers such as resistant starch (RS), inulin-type fructans and pectin-based matrices can resist immediate digestion by human amylases and proteases, which is useful for protecting ingredients until they reach the colon. For instance, resistant starch or certain fibers can carry probiotic bacteria or polyphenols through the upper gastrointestinal tract and only break down under enzymes produced by colonic microbiota. This ensures site-specific delivery in the colon for nutraceuticals that act on the gut microbiome. Wang et al. (69) demonstrated that microencapsulating probiotics within a RS matrix significantly enhanced their survival under harsh gastric acid and bile salt conditions, while facilitating targeted delivery to the colon. The RS-encapsulated probiotic formulation (RS-Pro) exhibited improved viability under simulated gastrointestinal conditions and showed microbiota-dependent degradation and site-specific release in mice, effectively alleviating chemotherapy-induced intestinal injury and dysbiosis. Tang et al. (70) summarized that natural biopolymers such as resistant starch and specific dietary fibers are resistant to enzymatic degradation in the small intestine but are selectively broken down by microbial enzymes in the colon. This property enables the targeted release of encapsulated polyphenolic compounds, facilitating site-specific delivery (70). Feng et al. (71) provided a comprehensive overview of various colon-targeted delivery systems, including resistant starch and natural biopolymers, emphasizing their shared mechanism of action: bypassing enzymatic degradation in the upper gastrointestinal tract and undergoing microbial enzyme-triggered breakdown in the colon to release bioactive compounds.

Importantly, these natural polymer nanocarriers align with the clean-label trend in functional foods. They offer a way to achieve “smart” delivery (triggered release, improved bioavailability) without resorting to synthetic materials. Through the 2010s and into the 2020s, such bio-based nanocarriers have been widely researched, and some have found use in products like protein-fortified drinks, encapsulated probiotics, and stabilized vitamin formulations. The diversification of nanoencapsulation strategies, ranging from advanced lipid nanoparticles (SLNs and NLCs) to nanoemulsions and biopolymer-based nanosystems, has greatly expanded the toolbox for nutraceutical formulation in this era.

#### 2.2.3 Advanced manufacturing and functional integration (recent trends)

In the most recent phase of this technological evolution, the focus has shifted to precision manufacturing techniques and multifunctional integration in nanoencapsulation systems, the preferred advanced nanoencapsulation techniques for major nutraceutical categories is shown in Table 1. One significant trend is the adoption of electrohydrodynamic fabrication methods- namely electrospinning and electrospraying – to create novel nutraceutical carriers. These techniques use high-voltage electric fields to process a solution of polymers (often loaded with nutraceutical actives) into nano-scale fibers or particles, respectively (72, 73). Unlike some conventional methods, electrospinning/electrospraying is typically conducted at ambient or low temperatures, making it especially suitable for heat-sensitive bioactives (for example, certain vitamins, antioxidants, or probiotic organisms that might be destroyed by high heat). The review of Mendes and Chronakis (74) supported this perspective: they highlighted that electrohydrodynamic (EHD) methods- including electrospinning and electrospraying- can be conducted at room temperature using aqueous solutions, without the application of heat, thereby preserving the viability and functional integrity of the live probiotic cells. The review by Bhushani and Anandharamakrishnan (75) also confirmed that electrospinning and electrospraying are non-thermal processes capable of generating nano- or microscale fiber and particle systems without compromising heat-sensitive compounds such as proteins and antioxidants. Moreover, Electrospinning produces ultra-fine nanofibers which can encapsulate nutraceutical compounds within or between polymer strands. The resulting fibrous mats have a very high surface-area-to-volume ratio and can be used as fast-dissolving delivery strips or as scaffolds that release nutrients slowly. Research has shown that electrospun nanofibers can stabilize poorly soluble compounds in an amorphous form, thereby enhancing their solubility, bioavailability and targeting of drug release (76). Rüzgar et al. (77) utilized HPMC/PEO-based electrospun nanofibers to encapsulate curcumin and observed a substantial improvement in its solubility. While free curcumin is virtually insoluble in water and pH 1.2 buffer, encapsulation increased its solubility to approximately 7.66 mg/L in water and 1.57 mg/L in acidic buffer. The study concluded that electrospinning is a promising approach for enhancing the solubility of poorly water-soluble compounds like curcumin (77). Electrospinning also allows controllable surface functionalization of fibers, meaning one can tune how the nutraceutical is released (rapidly or in a sustained manner) by adjusting fiber composition and morphology (78, 79).

**Table 1:** Preferred advanced nanoencapsulation techniques for major nutraceutical categories.

Nutraceutical category	Preferred technique(s)	Rationale (process/functional fit)	Typical outcomes	Reference
Heat-sensitive vitamins (e.g., vitamin C, B12), proteins, probiotics	Electrospinning, electrospraying, microfluidics, nanoliposomes	Non-thermal/low-temperature processing; mild solvent systems; biocompatible lipid-bilayer encapsulation of water-soluble actives	Enhanced retention of labile compounds; improved thermal and oxidative stability; protected bioactivity	(72–75, 82–84)
Poorly water-soluble polyphenols/antioxidants (e.g., curcumin, carotenoids)	Electrospinning (nanofiber amorphization), Electrospraying (dense nanospheres), nanoemulsion, nanoprecipitation, nanoliposomes	High-surface-area colloids and carriers enhance dispersibility; surfactant-stabilized oil droplets and polymer nanoparticles solubilize lipophilic actives	Improved dissolution and bioavailability; controlled release; suppression of crystallization and oxidation	(76–84)
Probiotics/enzymes (requiring powder form and food compatibility)	Electrospraying (dry powder nanocapsules), Microfluidics (multi-core microcapsules)	Rapid solvent evaporation for tight entrapment; multi-core/core–shell structures enhance acid and bile salt tolerance	Stable dry powders at ambient temperature; improved storage stability; higher gastrointestinal survival rates	(80–84)
Multi-component formulations (e.g., Omega-3 + Vit D3; polyphenol combinations)	Co-encapsulation (often with microfluidics), Nanoemulsion, Nanoliposomes	Simultaneous incorporation of multiple actives in hybrid carriers; synergistic interaction of co-encapsulated compounds	Synergistic efficacy, taste masking, enhanced stability and synchronized release	(85–89)
Precision/programmable delivery (stimuli-responsive, site-specific)	Microfluidics (monodisperse, multi-compartment/Janus/core–shell systems)	Highly programmable particle architectures; supports incorporation of stimuli-responsive materials	Narrow size distribution; tunable release triggers; targeted site-specific delivery	(85, 86)
Probiotics and enzymes (GI-targeted delivery)	Hydrogels; Nanogels	Three-dimensional polymer networks swell and protect actives in gastric conditions; pH-sensitive release in intestine	Enhanced survival of encapsulated probiotics/enzymes through the GI tract; site-specific (intestinal) release; improved stability and mucoadhesion	(265, 266)

Electrospraying, on the other hand, uses a similar setup but with lower solution viscosity, resulting in the formation of nano- or microspheres instead of fibers (80, 81). Electrosprayed particles can serve as dry powder nanocapsules that encapsulate nutraceutical ingredients with high efficiency. Because the solvent evaporates quickly during the electrospraying process, the active compounds are effectively “freeze-dried” into the particle matrix, often preserving sensitive ingredients without exposure to harsh conditions (82, 83). This method offers excellent control over particle size and uniformity, and it’s *scalable* – useful for producing stable powdered nutraceuticals (like a dry probiotic or enzyme formulation) that can be easily blended into foods or capsules, the reviews of Jayaprakash et al. (82) and Raval et al. (84) proved this view. Both electrospinning and electrospraying represent advanced manufacturing approaches that provide precise control over nanocarrier architecture, enabling better performance in delivery (e.g., faster dissolution or more complete release of actives at the target site).

Likewise, microfluidic-based fabrication is emerging as another advanced technique for creating nutraceutical carriers. Microfluidic devices use tiny channels to precisely control fluid flows, enabling the production of emulsions, nanoparticles, or microcapsules with uniform sizes and well-defined structures (85). This high level of control can improve the dispersibility, stability, and bioavailability of encapsulated bioactives. Furthermore, like EHD methods, microfluidic processes are typically carried out under mild, ambient conditions, which helps avoid degrading heat-sensitive compounds. Notably, microfluidics also opens the door to novel multi-compartment architectures (e.g., core-shell or Janus particles) by merging multiple streams into one microencapsulated droplet. For example, Zhao et al. (86) employed a microfluidic Y-junction device to produce “Yin-Yang” dual-core microcapsules that encapsulated two different probiotic species in separate compartments within one capsule. This multi-core design maintained the viability of both probiotics (each core provided a protected micro-environment) and leveraged their synergistic interactions- the microcapsules exhibited enhanced acid resistance and *in vivo* efficacy, significantly reducing intestinal inflammation and improving metabolic markers in an animal model. Such results demonstrate how microfluidic assembly can integrate multiple functional ingredients into a single delivery vehicle, achieving effects that would be difficult to accomplish with single-component carriers. However, a current limitation of microfluidic approaches is scalability: producing large batches of nanocarriers via microfluidics is challenging due to the low throughput of individual microchannels and issues like microchannel clogging (85). As a result, microfluidic techniques are presently used mainly as research tools to fabricate prototype delivery systems and to study how specific particle features (size, composition, and morphology) influence nutraceutical performance, rather than for mass production. Even so, ongoing advances (such as parallelizing many microchannels in tandem) may eventually improve the throughput, enabling wider application of this technology in nutraceutical manufacturing.

Another hallmark of recent nanoencapsulation research is co-encapsulation and multi-component delivery systems. Instead of delivering a single active ingredient, scientists are formulating nanocarriers that can hold and release multiple bioactive compounds together. The rationale is that many nutraceuticals could work in synergy: by delivering them simultaneously at the right ratio, one might amplify health benefits or address multiple targets in the body. Co-encapsulation platforms have been explored for combinations such as probiotics with prebiotics (synbiotics), various antioxidant phytochemicals delivered together, or fatty acids combined with vitamins. Liu et al. (87) noted in their review that co-encapsulation systems can effectively mask the astringency of different bioactive compounds, enhance their stability and bioavailability, and maximize their biological effects through synergistic interactions. Shakeri et al. (88) investigated the co-encapsulation of omega-3 and vitamin D3 in beeswax-based solid lipid nanoparticles and found that this system effectively masked the oily taste, protected the sensitive nutrients from degradation, and ensured their synchronized release at the absorption site. More importantly, co-delivery can produce synergistic effects- the combined efficacy is greater than each component alone- by synchronizing their bioavailability (89). For example, a study on the co-encapsulation of astaxanthin and kaempferol demonstrated that, compared to nanoparticles encapsulating astaxanthin alone, the combined formulation exhibited significantly stronger antioxidant activity and lipid-regulating gene expression in RAW264.7 and HepG2 cells, indicating a pronounced synergistic effect (90). Wei et al. (91) demonstrated that co-encapsulation exerts a synergistic effect in enhancing the photothermal stability of β-carotene and curcumin-loaded microparticles. In addition, Liu et al. (87) noted in their review that co-encapsulation systems can “mask astringency of different bioactive ingredients and enhance their stability and bioavailability, as well as to maximize the biological function of bioactive ingredients with synergistic effect.” Importantly, they emphasized that synchronized release plays a crucial role in achieving these synergistic effects, with combined formulations showing markedly superior efficacy compared to individual components- a point that deserves particular attention (87). These multi-component nanocarriers are still largely in the research phase, but they represent a push toward more holistic nutraceutical interventions- delivering a “cocktail” of beneficial agents in a precisely engineered vehicle.

In summary, nanoencapsulation in nutraceuticals has evolved from simple protective concepts into sophisticated, multifunctional delivery systems. Early work in the 1990s demonstrated that nanoscale carriers such as liposomes and SLNs could shield sensitive nutrients and improve their delivery. By the early 2000s, innovations including NLCs, nanoemulsions, and biopolymer-based nanoparticles expanded the range of available strategies and enhanced key performance metrics such as stability, payload capacity, and bioavailability. More recently, advances in manufacturing techniques and carrier design have given rise to intelligent nanosystems that are capable of co-delivering multiple bioactives or releasing their payload in response to specific physiological cues, which are well aligned with the demands of precision nutrition and complex health applications. This progression from basic protective shells to highly engineered, responsive nanocarriers reflects a steady refinement of both materials and methods, opening new avenues for maximizing the functional impact of bioactive compounds and improving health outcomes through next-generation nutritional interventions.

## 3 Functional roles and mechanistic basis of nanoencapsulation in nutraceuticals

**Figure 2:**
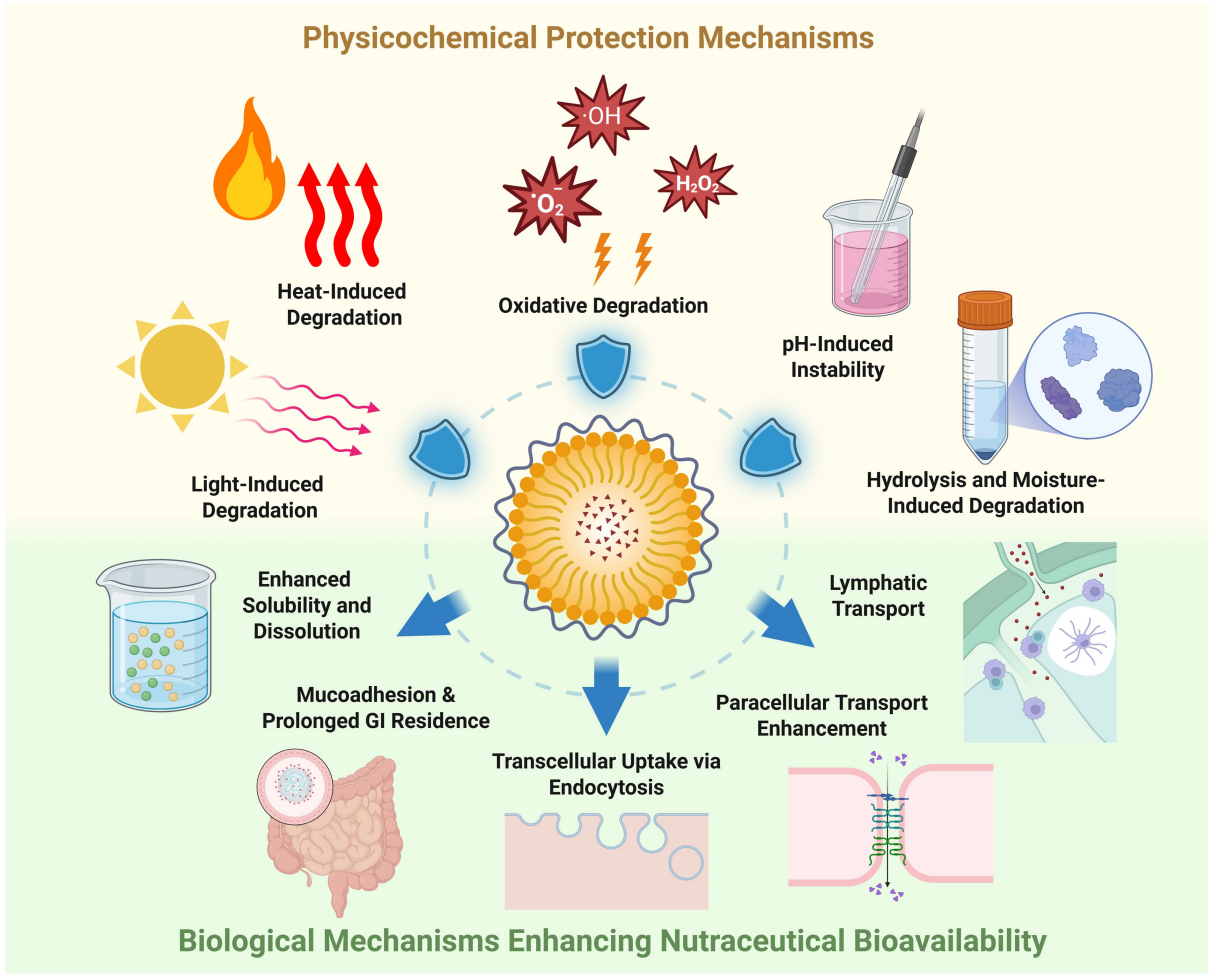
The physicochemical and biological mechanisms through which nanoencapsulation enhances the stability, solubility, bioavailability, and targeted delivery of bioactive compounds. Physicochemical protection: Shielding bioactive compounds against environmental stressors including light, heat, oxidation, pH fluctuations, and moisture/hydrolysis. Biological mechanisms: enhancing gastrointestinal performance by improving solubility and dissolution, promoting mucoadhesion and prolonged GI residence, facilitating transcellular uptake, enabling paracellular transport, and exploiting lymphatic pathways to bypass first-pass metabolism. Created with BioRender.com.

In the preceding section, we examined the technological evolution of nanoencapsulation in nutraceuticals, tracing its trajectory from conceptual foundations and early prototypes- such as liposomes and solid lipid nanoparticles- to contemporary multifunctional and stimuli-responsive delivery platforms. That discussion emphasized how advances in materials science, fabrication techniques, and carrier design have progressively expanded the capabilities of nanoencapsulation systems. Building on this historical and technological context, the present section shifts focus from how these systems are developed to why they matter in practice, by dissecting the core functional roles that underpin their value in nutraceutical applications. Specifically, the physicochemical and biological mechanisms through which nanoencapsulation enhances the stability, solubility, bioavailability, and targeted delivery of bioactive compounds (shown in Figure 2) are explored, as understanding these mechanisms is essential for translating engineering innovations into effective, evidence-based nutritional interventions.

### 3.1 Physicochemical protection mechanisms

Nanoencapsulation of nutraceuticals (vitamins, polyphenols, carotenoids, omega-3 s, etc.) has been widely shown to enhance their physicochemical stability and shelf-life. By entrapping bioactive compounds in nanocarriers (liposomes, lipid nanoparticles, nanoemulsions, biopolymer nanoparticles, etc.), researchers have observed protection against environmental stressors such as light, heat, oxygen, pH extremes, and moisture. Below we summarize recent peer-reviewed studies demonstrating how nanoencapsulation confers stability to nutraceuticals under various degradation conditions.

#### 3.1.1 Protection against light-induced degradation

Exposure to light (especially UV) can rapidly degrade light-sensitive nutraceuticals like curcumin and carotenoids. Nanocarriers can act as physical shields or UV filters that improve photostability.

*Curcumin*: Encapsulation of curcumin in a corn starch/OSA-starch-whey protein emulsion gel significantly increased its resistance to light. In the study of Zhan et al. (92), curcumin-loaded emulsion gels with high oil content showed “greater protection against light irradiation” compared to less structured formulations. Encapsulated curcumin’s stability under light was markedly improved, confirming that the nano-scale starch-protein matrix can absorb or scatter deleterious light.

*β-carotene*: Nano/microencapsulation dramatically extends the photostability of carotenoids. The study of Drosou and Krokida (93) comparing spray-dried vs. freeze-dried β-carotene encapsulates found that the spray-dried particles (pullulan-WPI wall) prolonged the half-life of β-carotene under UV–Vis light to 336 h, versus 102 h for the other encapsulate. This indicates that an optimized nanoencapsulation method can triple the photostability of β-carotene relative to less protected forms. Other work on Pickering emulsions has similarly shown that encapsulating β-carotene in a protein/polysaccharide particle system enhances its stability under UV exposure compared to free β-carotene (94). The spray-dried vs. freeze-dried β-carotene encapsulates exemplify how formulation method impacts outcomes. Spray drying with a pullulan–WPI wall produced a denser, more uniform matrix around the β-carotene, which better blocked UV–Vis light, whereas freeze drying yielded a more porous structure offering weaker protection. This structural difference explains the 3× longer half-life of β-carotene in the spray-dried powder. In essence, nanoencapsulation methods that create a tight, continuous barrier around the active compound (as with the starch-protein gel for curcumin or the pullulan–WPI microcapsule for β-carotene) provide superior photoprotection by absorbing or scattering incoming UV radiation before it can reach the nutraceutical. Other studies confirm this trend: for instance, protein–polysaccharide Pickering particles also significantly improved β-carotene’s light stability relative to non-encapsulated carotene. These comparisons underscore a clear mechanism- the more cohesive and opaque the encapsulating matrix, the greater the shielding effect- and they reconcile findings across different formulations. Any apparent discrepancies between studies can often be attributed to matrix quality: when an encapsulation matrix is optimized (fine particle size, low porosity, strong light absorbance), photodegradation is minimized, whereas suboptimal encapsulates offer less protection. Thus, seemingly disparate results in the literature actually highlight the same principle: robust nanocarriers consistently mitigate light-induced nutrient loss, and variations in effectiveness are due to how well each carrier is engineered as a light barrier.

#### 3.1.2 Protection against heat-induced degradation

Heat during processing or storage can denature vitamins and oxidize unsaturated compounds. Nanoencapsulation often improves thermal stability by insulating the core and preventing direct heat exposure or chemical breakdown.

*Vitamin C*: Ascorbic acid is notoriously heat-labile, but encapsulation raises its thermal tolerance. For example, spray-dried alginate/gum arabic microcapsules loaded with vitamin C increased its decomposition temperature to 188 °C, well above typical food processing temperatures (95). Encapsulated vitamin C retained ~90% of its content after 60 days at room temperature, whereas unprotected vitamin C would degrade much faster (96). This demonstrates that a polymeric nano/microcapsule can shield vitamin C from thermal breakdown and prolong its shelf-life.

*Carotenoids (β-carotene, lutein)*: Encapsulation also guards lipid-soluble nutraceuticals during heat treatment. A study of Lim and Roos (97) showed that trehalose/maltodextrin glassy nanoparticles “effectively protected encapsulated lutein and all-trans-β-carotene during storage at 35 °C, 50 °C, and 65 °C”. In fact, a spray-dried microencapsulated Chlorella extract retained about 65% of its lutein after storage at elevated temperature- roughly double the retention of the unencapsulated extract (98). Such data confirm a real improvement in heat stability: encapsulated carotenoids survived pasteurization-like temperatures far better than free carotenoids, which often isomerize or oxidize when heated.

#### 3.1.3 Protection against oxidative degradation

Many nutraceuticals undergo oxidative rancidity or chemical oxidation in the presence of air. Nanoencapsulation provides an oxygen barrier and can incorporate antioxidants to drastically slow oxidation. The summary of nanoencapsulation strategies that mitigate oxidative degradation was shown in Table 2.

**Table 2:** Summary of nanoencapsulation strategies that mitigate oxidative degradation.

Target bioactive/instability driver	Encapsulation approach	Materials	Mechanistic basis of oxidation control	Performance improvement (vs. free bioactive)	Reference
Omega-3 fatty acids (highly unsaturated; prone to peroxidation)	Protein nanocapsules (electrospraying)	Zein vs. kafirin (corn proteins)	Dense hydrophobic shell limits O₂ diffusion; higher encapsulation efficiency reduces exposed surface	Zein nanocapsules showed ~3.3 × greater oxidative stability than kafirin; high omega-3 retention during storage	(99)
	Spray-dried microparticles	Gelatin, maltodextrin, modified starch, whey protein isolate	Glassy carbohydrate–protein wall acts as O₂ barrier; isolates oil at interface	Encapsulated powders: PV < 10 meq/kg after 35 d vs. 30–40 meq/kg for free oil	(93, 100)
	Nanoemulsion (co-loading antioxidants)	Fish oil + curcumin/resveratrol	Co-encapsulated antioxidants scavenge radicals inside droplets; synergistic protection	Markedly lower peroxide formation than plain fish-oil emulsions	(101)
Vitamin E (self-oxidation over storage/light)	Electrospun nanofibers	Zein (corn protein)	Continuous hydrophobic fiber matrix isolates vitamin E from pro-oxidants	~60% encapsulation efficiency; substantially enhanced oxidative stability	(102)
	Whey-protein colloidal particles	Whey protein isolate	Protein shell reduces O₂ access; improved dispersibility minimizes interfacial oxidation	Significantly enhanced water dispersibility and oxidation resistance	(103)
	PCL/gelatin nanofibers	PCL (polycaprolactone) + gelatin	Polymer blend barrier with low O₂ permeability and radical access	Protective effect: reduced oxidative stress of vitamin E during storage	(104)
Anthocyanins (polyphenolic pigments; color/activity loss by oxidation)	Biopolymer/lipid encapsulation (nano/micro)	Pullulan + WPI; chitosan/alginate, etc.	Physical O₂ barrier; controlled release; stabilization of labile pigment form	Encapsulated anthocyanins show higher oxidative/thermal stability vs. free; preserved antioxidant activity	(105, 106)
Broad polyphenols (e.g., catechins, resveratrol; oxidation-sensitive)	Protein/polysaccharide matrices or nanoemulsions	Various food-grade proteins/polymers	Barrier to O₂ and radicals; sustained release; interfacial stabilization	Slower oxidation rates; prolonged shelf-life and maintained bioefficacy	(107)

PV, peroxide value; WPI, whey protein isolate; PCL, poly(ε-caprolactone).

*Omega-3 fish oil*: Polyunsaturated omega-3 fatty acids are highly prone to oxidation (leading to off-flavors and nutrient loss). The study of Rahmani-Manglano et al. (99) denoted that the encapsulation of fish oil by electrospraying using both kafirin or zein as wall materials protected fish oil from oxidation. Notably, zein-based nanocapsules demonstrated 3.3-fold greater oxidative stability compared to kafirin-based nanocapsules, a difference that aligns with their higher oil encapsulation efficiency (99). This implies that a well-designed nano-shell can limit oxygen access to the oil core. Likewise, spray-drying fish oil with appropriate wall materials (e.g., gelatin, maltodextrin, modified starch or whey protein) yields microcapsules that significantly retard oxidation. Encapsulated fish oil powders exhibit much lower peroxide formation during storage (peroxide value <10 meq/kg after 35 days) compared to unencapsulated oil (which can exceed 30–40 meq/kg) (93). In effect, a well-designed microcapsule shell greatly extends the oil’ s shelf-life by limiting oxygen access to the lipid core (100). Instead of oxygen-impermeable walls alone, another strategy is co-loading antioxidants to sacrificially consume oxygen. For example, co-encapsulating curcumin or resveratrol with oils suppresses oxidation. Xiao and Ahn (101) showed that fish oil nanoemulsions co-encapsulated with curcumin and resveratrol exhibited much lower peroxide formation than plain fish oil emulsions. The nanocarrier both isolated the oil and allowed the polyphenols to neutralize free radicals, effectively preventing oxidative degradation of the omega-3.

*Vitamin E*: Although vitamin E is an antioxidant, it is itself susceptible to degradation when exposed to oxygen and light over time. Nanoencapsulation can preserve vitamin E’s activity by shielding it from ambient oxygen. Researchers have developed protein-based nanocarriers (e.g., electrospun zein fibers and whey proteins) that form a dense, uniform barrier around vitamin E, protecting it from oxidative conditions. A study of Mishra et al. (102) achieved nearly 60% encapsulation efficiency for vitamin E in corn-zein nanofibers (i.e., ~60% of the added *α*-tocopherol was retained in the fiber matrix). These zein fibers tend to be nanometric in diameter (hundreds of nm) and provide a continuous, uniform matrix that isolates vitamin E from pro-oxidant factors (102). Kong et al. (103) reported that the water dispersibility and resistance to oxidation of vitamin E were “significantly enhanced” by forming complexes with whey proteins. In essence, the whey protein shields the vitamin from pro-oxidant exposure similarly to zein, and also improves its solubility in functional beverages or supplements. Dairy proteins have the added advantage of amphiphilicity, allowing them to interact favorably with the hydrophobic vitamin E and form a protective colloidal shell. In addition, a variety of polymeric and protein-based nanostructures have thus been explored for stabilizing vitamin E. For example, Kalantary et al. (104) developed PCL/gelatin nanofiber mats incorporating vitamin E to provide protection against oxidative stress.

*Polyphenols*: Plant polyphenols are another class of nutraceuticals prone to oxidation (which can diminish their color and bioactivity). For example, anthocyanins- the antioxidant pigments in berries and grapes- degrade rapidly when exposed to oxygen, light, or heat. Encapsulating such polyphenols in nanoscale carriers provides a functional oxygen barrier that significantly enhances their stability (105). Studies report that encapsulated anthocyanins have much higher oxidative and thermal stability compared to their non-encapsulated counterparts (106). By isolating polyphenolic compounds from pro-oxidant environmental factors, nanoencapsulation helps preserve their nutritional efficacy and prevents the loss of antioxidant activity. In general, nano-formulation of polyphenols is aimed at protecting these compounds from degradation while also allowing for controlled, sustained release of the actives (107). This means consumers can obtain the intended health benefits of polyphenols (such as catechins, resveratrol, or curcumin) over a longer shelf-life and through targeted delivery, without the compounds prematurely oxidizing.

Each of these examples illustrates that a well-designed nanoencapsulation system can limit oxygen access to sensitive nutraceutical cores, thereby protecting them from oxidative degradation. This strategy is crucial for preserving the quality, potency, and shelf-life of oxygen-sensitive nutrients in functional foods and supplements. The choice of encapsulating material (proteins, polysaccharides, lipids, etc.) and method (electrospraying, spray-drying, liposomes, and emulsions) can be tailored to the nutraceutical in question, but the overall outcome is a significant reduction in oxidation rate and improved stability of the active compound in ambient conditions. By slowing down oxidation, nanoencapsulation helps ensure that consumers receive the full nutritional and organoleptic benefits of ingredients like omega-3 oils, vitamins, and antioxidants throughout a product’s intended shelf-life.

#### 3.1.4 Protection against pH-induced instability

Bioactives can degrade or precipitate in the acidic stomach or be unstable in alkaline conditions. pH-responsive nanoformulations can protect compounds through the stomach and release them in the intestine, thereby preventing pH-triggered decomposition.

*Vitamin C:* Free ascorbic acid tends to degrade in the acidic gastric environment. Encapsulation in pH-sensitive carriers like chitosan can delay its release until higher pH is encountered. In a study of Alishahi et al. (108), only ~30% of vitamin C encapsulated in chitosan nanoparticles was released in a simulated gastric solution (pH ~ 2), whereas over 75% was released in simulated intestinal conditions (pH ~ 7). This indicates that the nanoparticle largely protected the vitamin from immediate acid attack, releasing the bulk of the dose later in the intestine. Such controlled release improves bioavailability and stability.

*Curcumin:* Curcumin is unstable in neutral-basic pH (degrading rapidly in intestinal conditions) and also poorly soluble. Complex coacervate microcapsules have been used to address this. A study of encapsulated curcumin in lysozyme-polysaccharide coacervate microcapsules and found that the microcaps “protected curcumin during the oral and gastric phases,” with 77–94% of the curcumin only released in the intestinal phase (109). In other words, negligible curcumin was lost in the low pH of stomach; most payload was delivered intact to the intestine. This validates that nanoencapsulation can prevent pH-induced breakdown (or premature release) by using coatings that remain intact in harsh stomach acid but dissolve at intestinal pH. In the study of Zhang et al. (110), shellac-curcumin nanoparticles made by a pH-cycle method exhibited good physicochemical stability and bioaccessibility. In shellac-coated zein core-shell microparticles, curcumin release was ~10% at pH 1.2, ~45% at pH 6.8, and showed burst release at pH 7.8, a clear enteric trigger.

*Anthocyanins:* As noted above in Section 3.1.3, nanoencapsulation substantially enhances anthocyanin stability. The chitosan/alginate capsules here also showed effective GI stability and targeted release. Complementary work with gellan-whey nanocomplexes reports similar gains in stability and bioaccessibility (111).

*β-carotene:* Emulsions stabilized by alginate-pectin-whey protein complexes showed enhanced storage stability and a GI sustained-release profile (limited gastric release, increased intestinal release), illustrating how protein-polysaccharide co-assemblies can act as enteric-like emulsifiers (112).

By tailoring encapsulant materials (e.g., enteric polymers, chitosan, alginate) that respond to pH, nutraceuticals can be shielded from both acidic and basic instability in the GI tract, ensuring they reach the absorption site without degradation.

#### 3.1.5 Protection against hydrolysis and moisture-induced degradation

Moisture and water can hydrolyze sensitive compounds and accelerate spoilage. Nanoencapsulation often involves drying bioactives into powders or encapsulating them in hydrophobic matrices, thereby reducing their exposure to moisture.

*Ascorbic acid and water activity:* Ascorbic acid in solution readily hydrolyzes to dehydroascorbic acid and further to diketogulonic acid, especially under heat or moisture. Encapsulation can keep ascorbic acid in a protected, low-moisture microenvironment. For instance, alginate/gum arabic microcapsules not only improved vitamin C’s heat tolerance, but also prevented moisture-driven degradation, as evidenced by ~90% vitamin C retention after 2 months storage at room conditions. By contrast, unencapsulated vitamin C would show extensive loss due to hydrolysis and oxidation over that time. This highlights that the capsule’s barrier to environmental humidity preserves the core compound (96).

*Hygroscopic nutraceuticals:* Many nutraceutical powders (e.g., certain amino acids, plant extracts) are hygroscopic and lose stability upon absorbing moisture. Encapsulation can lower a powder’s hygroscopicity. Table 3 summarizes food-grade encapsulation materials that reduce moisture uptake in hygroscopic nutraceutical powders, outlining their barrier rationale, common fabrication formats, suited actives, practical formulation notes, and key references. Beyond the choice of wall composition, the manufacturing route strongly modulates moisture uptake. Spray-drying at higher total solids and with lower-DE maltodextrin typically yields glassy, low-porosity shells, which reduce moisture uptake, whereas lower solids content or high inlet humidity tends to produce more porous, sticky powders with higher hygroscopicity (113, 114). Freeze-drying often produces thicker, less-collapsed matrices that show lower water sorption at the same composition, although residual porosity may remain if secondary drying is not applied to desorb bound water (115, 116). Electrospinning/electrospraying can create dense nanofibrous or core-shell particles with hydrophobic outer layers (e.g., zein or ethyl cellulose) that function as effective moisture barriers (supported by general understanding of barrier performance in such systems) (117). Spray-chilling or lipid-based encapsulation (e.g., carnauba wax or beeswax) forms crystalline shells that are inherently moisture-resistant (common in lipid encapsulation literature) (118, 119). Layer-by-layer or complex-coacervation coatings (e.g., chitosan/alginate or protein-polysaccharide matrices) provide thin, conformal moisture shields, and maintaining low ionic strength during assembly and applying mild post-curing generally improves barrier integrity (a principle well established in colloid/film coatings). In practice, hygroscopicity reflects the combined influences of composition and structure- including solids content, particle size, glass transition temperature, porosity, and shell continuity- rather than formulation alone (120).

**Table 3:** Encapsulation materials and coatings that mitigate moisture uptake (hygroscopicity) in nutraceutical powders.

Encapsulation material (wall/coating)	Moisture-barrier rationale	Typical format/process	Suited bioactives	Relative barrier performance	Practical notes for formulators	Reference
Zein (corn protein)	Intrinsically hydrophobic; low water vapor transmission; dense protein network limits diffusion	Electrospun/electrosprayed fibers or capsules; cast films; spray-dried particles	Lipophilic vitamins (E, D), carotenoids, essential oils	High	Works well as an outer shell to suppress surface moisture; combine with plasticizers minimally to keep barrier strong	(267, 268)
Zein + EC hybrid	Synergistic hydrophobic matrix; EC adds additional water resistance	Electrospinning/electrospraying; film-forming	Lipophilic antioxidants, flavors	Very high	Hybrid shells show lower water uptake than zein alone; tune zein: EC ratio to balance brittleness vs. barrier	(269)
Ethyl Cellulose	Water-insoluble moisture barrier	Core-shell microcapsules (coaxial electrospraying); film coatings	Oils (omega-3), vitamin A/E esters, volatile flavors	High	Effective core or outer coat; combine with OSA-starch or proteins for mechanical strength	(270)
Shellac (food-grade resin)	Highly hydrophobic; low WVTR; robust film-forming	Particle/granule coatings; over-coats	Curcumin, carotenoids, fat-soluble vitamins	Very high	Natural, PFAS-free moisture/grease barrier; avoid strong alkali	(271)
Carnauba/Beeswax (waxy lipids)	Crystalline lipid shell repels water; fills surface pores	Melt dispersion; spray-chilling; lipid microcapsules; coatings	PUFA oils, flavors, botanicals	High	Excellent moisture resistance; consider melting point vs. process; may slow release	(272, 273)
OSA-modified starch	Amphiphilic granules create compact shells; surface less polar	Spray-drying; core-shell with EC/proteins	Emulsified oils, vitamin A/E, carotenoids	Moderate	Reduces stickiness and moisture pickup in powders when solids are high and drying setpoints optimized	(274)
MD + GA	Dense carbohydrate glass; GA improves film integrity/interfacial packing	Spray-drying (common)	Oils, flavors, polyphenol extracts	Moderate	Lower DE MD (higher MW) and higher wall-solids generally lower hygroscopicity; optimize MD: GA for flow ability	(275, 276)
Inulin + WPI	Protein-fiber network forms less porous, glassy matrices	Freeze-drying (robust walls), spray-drying	Carotenoids, plant extracts, vitamins	High	Inulin reduces stickiness and moisture sorption; freeze-dried matrices often show lower hygroscopicity at equivalent solids	(277)
Pullulan + WPI	Tight film-forming polysaccharide with protein co-matrix; reduced pore connectivity	Spray-drying or freeze-drying	β-carotene, sensitive pigments	High	Pullulan aids barrier/film strength; choose process based on target porosity and water activity of storage	(93)
Chitosan/alginate (polyelectrolyte complexes)	Ionic crosslinks yield compact shells; reduced water ingress at low aw	Complex coacervation; LbL coatings	Polyphenols, vitamin C (as salts/derivatives)	High	Good as thin LbL moisture shields around emulsions/particles; keep ionic strength low during assembly	(278, 279)
Pectin/Gellan + Proteins	Protein-polysaccharide co-gels reduce capillary condensation	Emulsion gels; spray- or freeze-drying	Anthocyanins, curcumin, mixed botanicals	Moderate	Effective in beverage concentrates and powders; adjust pH near biopolymer complexation window for densest network	(280, 281)

OSA, octenyl succinic anhydride; EC, ethyl cellulose; WVTR, water-vapor transmission rate; PUFA, polyunsaturated fatty acid; MD, maltodextrin; GA, gum arabic; DE, dextrose equivalent; MW, molecular weight; WPI, whey protein isolate; LbL, layer-by-layer.

### 3.2 Biological mechanisms enhancing nutraceutical bioavailability

Nanoencapsulation offers several mechanisms to enhance bioavailability: it improves aqueous solubility, enhances mucosal adhesion, promotes endocytosis-mediated uptake, facilitates lymphatic transport, and allows for sustained release, all of which contribute to more effective delivery. Recent studies exemplifying these mechanisms are summarized below.

#### 3.2.1 Enhanced solubility and dissolution

One of the most fundamental advantages of nanoencapsulation is the significant enhancement of solubility and dissolution rate for poorly water-soluble nutraceuticals. Many bioactive compounds such as curcumin, resveratrol, and carotenoids exhibit pronounced lipophilicity, which limits their dispersion in the aqueous gastrointestinal environment and leads to extremely low bioavailability. Nanoscale delivery systems address this barrier through several complementary mechanisms.

*Increased apparent solubility:* By entrapping hydrophobic molecules within nanocarriers, the actives are dispersed at the molecular or colloidal level, preventing aggregation and crystallization. For instance, curcumin encapsulated in lipidic nanoconstructs (CLEN) displayed a 1.4 × 10^6-fold increase in aqueous solubility, which translated into a nearly 70-fold improvement in oral bioavailability compared with free curcumin (40). This dramatic enhancement highlights the ability of lipid-based nanosystems to maintain bioactives in a pseudo-dissolved state that is readily accessible for absorption.

*Particle size reduction:* Shrinking the particle size of nutraceutical carriers into the nanometer range drastically increases the surface area-to-volume ratio, accelerating dissolution into intestinal fluids. A study demonstrated that reducing the mean droplet diameter of an oily nutraceutical formulation from ~330 nm to ~90 nm resulted in a three-fold increase in oral bioavailability (121). The enhanced absorption was attributed to faster dissolution kinetics and improved diffusion of the nanosized droplets across the unstirred water layer adjacent to the intestinal epithelium.

*Surfactant-mediated stabilization:* In addition to particle size reduction, the incorporation of amphiphilic surfactants and stabilizers plays a pivotal role in enhancing the solubilization of lipophilic nutraceuticals. By lowering interfacial tension and improving wettability, surfactants facilitate the formation of small, stable nanodroplets or mixed micelles that markedly increase apparent solubility and accelerate dissolution rates in gastrointestinal fluids (122–125). Beyond the initial dispersion, surfactants sustain solubilization during intestinal transit by stabilizing emulsified droplets, inhibiting aggregation or Ostwald ripening, and promoting the formation of bile salt-phospholipid mixed micelles that maintain bioactives in a solubilized state (126–128). This stabilization effect is further reinforced by their capacity to sustain supersaturation and suppress precipitation or recrystallization, thereby prolonging the time window in which bioactives remain available for absorption (129). Surfactants such as d-*α*-tocopheryl polyethylene glycol 1,000 succinate (TPGS) and polysorbate 80 can inhibit intestinal efflux transporters, thereby overcoming efflux-limited absorption and further improving cellular uptake (130). Collectively, these mechanisms establish and maintain a favorable concentration gradient across the intestinal epithelium, which has been corroborated in both *in vitro* digestion models and *in vivo* studies demonstrating significantly higher oral bioavailability of hydrophobic nutraceuticals such as curcumin, carotenoids, and vitamin E when delivered in surfactant-stabilized nanoformulations.

#### 3.2.2 Mucoadhesion and prolonged GI residence

Many biopolymer-based nanocarriers (e.g., chitosan, alginate, pectin) exhibit mucoadhesive properties – they can electrostatically bind or form hydrogen bonds with the mucus gel layer that coats the gastrointestinal epithelium (131). By adhering to mucus, such nanoparticles are retained longer at the absorption site instead of being quickly swept away by peristalsis. This prolonged residence time allows more of the encapsulated nutraceutical to be released and absorbed *in situ*. For instance, an oral insulin loaded in chitosan-coated nanoparticles showed significantly extended retention on the intestinal mucosa and even penetrated into the underlying tissue, which translated to improved insulin absorption compared to non-encapsulated insulin (132). Generally, mucoadhesive nanocarriers create an “intimate contact” with the gut wall, increasing the local concentration gradient and time for absorption of the bioactive compound.

Nanodelivery systems such as gastroretentive floating beads, mucoadhesive lipid nanoparticles and porous silica-based carriers can prolong gastric residence, which is beneficial for actives absorbed in the upper small intestine and for synchronizing release with digestive processes in the duodenum. Lipid-based liquid crystalline nanocarriers such as cubic-phase glyceryl monooleate systems were found to swell and form a viscous gel in the stomach, exhibiting both mucoadhesion and sustained release. This combination increased the gastric retention time and subsequently enhanced the oral bioavailability of payloads like piperine (a poorly soluble nutraceutical) in one study (133). Prolonged gastric retention may protect acid-labile compounds by keeping them encapsulated until release in the higher-pH intestine, and it can synchronize the release of lipophilic nutrients with the onset of fat digestion/absorption processes in the duodenum, thereby improving uptake efficiency.

#### 3.2.3 Transcellular uptake via endocytosis

Unlike free molecules which must diffuse across cell membranes or tight junctions, nanoparticles can be actively taken up by intestinal epithelial cells (enterocytes) via endocytosis. Cells internalize nanocarriers through multiple energy-dependent pathways- including clathrin-mediated endocytosis, caveolae/lipid-raft mediated uptake, and micropinocytosis- engulfing the nanoparticle into vesicular compartments (134, 135). These vesicles can then transcytose across the cell and release the nutraceutical on the basolateral side (into systemic circulation or lymph). By leveraging these native uptake mechanisms, nanoencapsulation enables even large or poorly permeable compounds to traverse the intestinal epithelium efficiently. For example, particles below ~100–200 nm can penetrate cells whereas larger microparticles cannot. In essence, the nanocarrier acts as a Trojan horse: it is ingested by the cell like a nutrient or particulate, carrying the bioactive payload with it. This mechanism has been observed to significantly increase absorption of macromolecules (e.g., peptides and polysaccharides) and lipophilic phytochemicals that ordinarily have minimal uptake. Furthermore, some nanocarriers are designed with specific ligands (such as vitamin B12 analogs or lectins) to actively target receptor-mediated endocytosis pathways, thereby boosting cellular uptake via nutrient transporter routes (132, 136).

#### 3.2.4 Paracellular transport enhancement

Another way nanoencapsulation can improve absorption is by transiently opening the tight junctions between intestinal epithelial cells. Materials like chitosan and certain surfactants are known to interact with proteins in the tight junction complex, causing a reversible relaxation of these intercellular seals (131). This enlarges the paracellular pores and allows normally non-permeable hydrophilic molecules to slip between cells into the bloodstream. Chitosan nanoparticles, in particular, have been shown to reversibly increase paracellular permeability in both *in vitro* cell monolayers and *in vivo* intestinal models. By widening the paracellular route, nanoencapsulation circumvents the size and polarity restrictions of the cell membrane, permitting enhanced uptake of vitamins, antioxidants, and peptides that would otherwise be too large or too polar to cross. It is important that such tight-junction opening is temporary and safe- studies indicate chitosan’s effect is short-lived and non-damaging, and it ceases once the nanoparticle moves on (137). In addition to chitosan, various absorption enhancers (e.g., certain bile salt derivatives or novel peptides) are being incorporated into nanocarriers to promote paracellular transport without lasting harm to the epithelium. This strategy effectively increases the fraction absorbed of nutraceuticals limited by poor membrane permeability.

In addition, nanocarriers can also overcome biological barriers like P-glycoprotein (P-gp) efflux pumps that actively expel many nutraceuticals and drugs back into the intestinal lumen. Surface-active excipients commonly used in nanoparticle formulations (for example, vitamin E TPGS or polysorbate-80) are known to inhibit P-gp and other efflux transporters, thereby reducing the pumped-out fraction of the bioactive (121, 138). By incorporating such excipients, nano-formulations ensure that once the nutraceutical permeates into enterocytes, it is less likely to be ejected back and more will reach systemic circulation. This synergistic effect combines the enhancement of influx through mucoadhesion and endocytosis with the reduction of efflux, thereby markedly shifting the balance toward greater net absorption.

#### 3.2.5 Lymphatic transport and first-pass circumvention

Lipid-based nanosystems (e.g., nanoemulsions, solid lipid nanoparticles, self-emulsifying delivery systems) can exploit the intestinal lymphatic transport route to bypass first-pass metabolism in the liver. Normally, dietary long-chain lipids and fat-soluble vitamins are assembled into chylomicrons within enterocytes and secreted into the mesenteric lymphatics. Encapsulating nutraceuticals in lipid carriers, particularly when long-chain triglycerides or other fats are used as core materials, allows them to be incorporated into chylomicrons together with dietary lipids (139–141). The chylomicrons then carry the nutraceutical via the lymphatic vessels, which drain into the thoracic duct and systemic circulation, largely skipping the hepatic portal vein. This means the compound avoids immediate exposure to metabolic enzymes in the liver, resulting in a higher bioactive fraction reaching the bloodstream intact (133). Numerous studies confirm that co-ingesting lipophilic nutraceuticals (curcumin, quercetin, carotenoids, etc.) with lipid-based nanocarriers or emulsified fats leads to increased lymphatic uptake and significantly greater bioavailability compared to non-nano or fat-free formulations (142–144). The lymphatic route also tends to be a capacity-unlimited pathway (in contrast to saturable portal absorption), which further supports improved systemic delivery of high-dose nutraceuticals when delivered via nanocarriers designed to target this pathway.

An alternative lymphatic entry mechanism is through Peyer’s patch M cells- specialized epithelial cells in gut-associated lymphoid tissue that sample particles and deliver them to immune cells in the intestinal lymphoid follicles. M cells are highly effective at phagocytosing particulate matter and transporting it across the epithelium into the interstitial fluid and local lymphatics (145). Nanoencapsulation can leverage this by either using particle sizes and surface characteristics that favor M-cell uptake or by functionalizing nanocarriers with M-cell-targeting ligands (such as specific lectins or invasin peptides). The M cell pathway allows nanocarriers to directly enter the mesenteric lymph nodes and subsequently the lymph circulation, again bypassing the liver’s first-pass filter (146, 147). For example, researchers have shown that decorating nanoparticles with a targeting ligand (like a lectin that binds M cells) led to higher nanoparticle transcytosis into Peyer’s patches and substantially increased oral bioavailability of encapsulated antigens and drugs (132). In the nutraceutical context, targeting M cells could enhance the absorption of larger bioactives (e.g., peptides or particulate nutraceuticals like probiotics) that benefit from a lymphatic uptake route. By channeling nutrients through intestinal lymphatics (whether via chylomicrons or M cells), nano-formulations thus minimize first-pass metabolic losses, leading to higher systemic levels of the active compounds.

## 4 Commercial applications

**Figure 3:**
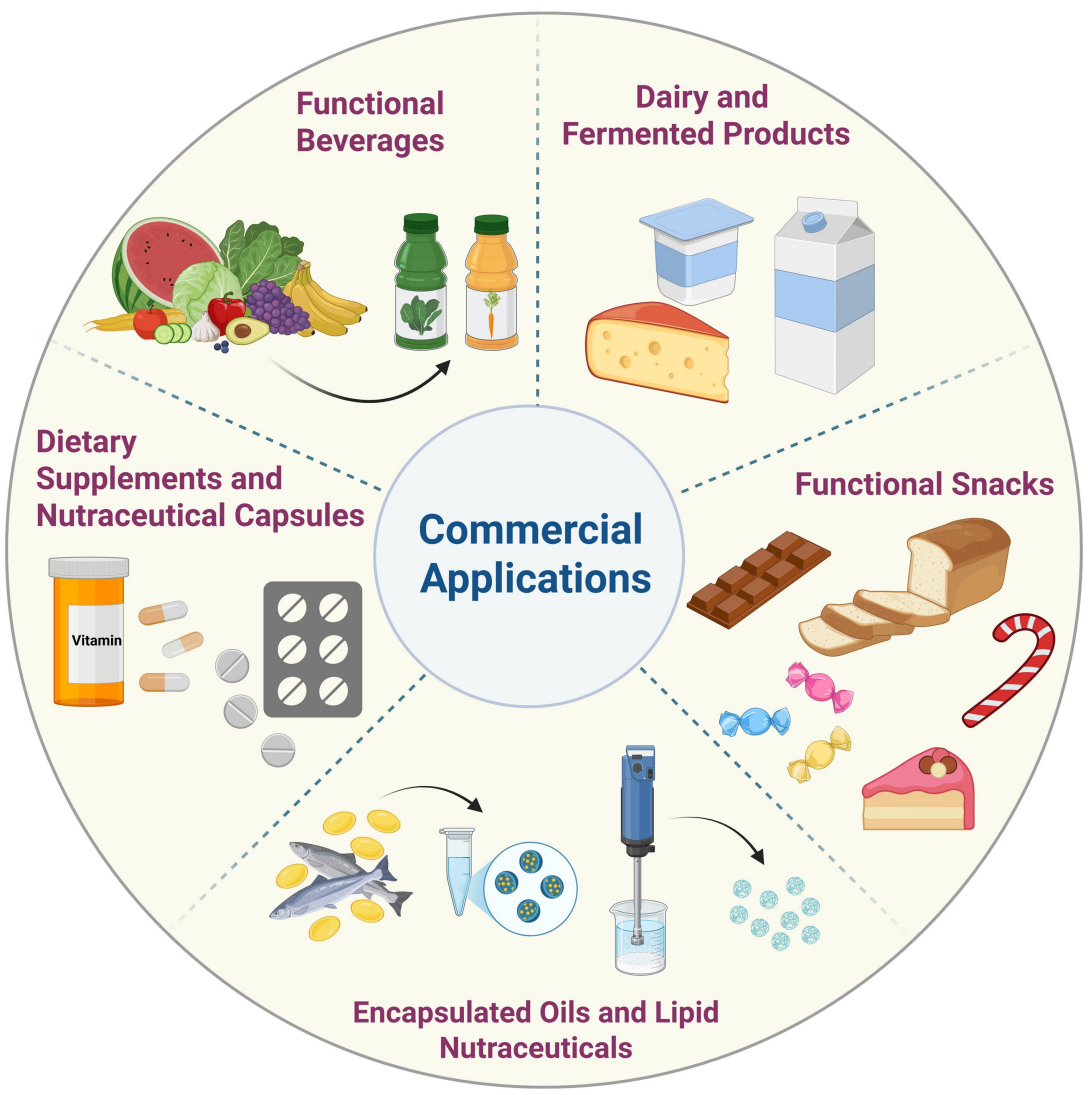
Commercial applications of nanoencapsulation in nutraceuticals and functional foods. Applications are categorized into five major sectors: functional beverages, dairy and fermented products, dietary supplements, functional snacks, and encapsulated oils. These examples demonstrate how nanoencapsulation technologies are moving from laboratory innovations to large-scale commercial deployment. Created with BioRender.com.

In recent years, nutraceutical and functional food industries have begun integrating nanoencapsulation technologies into commercial products on a significant scale. Enhanced bioavailability and stability offered by nano-delivery systems have driven a surge of nano-enabled product launches (examples are shown in Figure 3, and the commercial applications of nanoencapsulated nutraceuticals in food products were list in Table 4). For instance, over 85 new nutraceutical products featuring nanotechnology were introduced in 2023, and approximately 68% of nutraceutical manufacturers are now investing in nano-delivery systems for ingredients like omega-3 fatty acids, antioxidants, and plant extracts (148). These innovations aim to address the longstanding challenges of poor solubility and low absorption of many bioactive nutrients. By packing vitamins, polyphenols, probiotics, and oils into nanocarriers (typically <100 nm), companies can protect sensitive ingredients through processing and digestion, mask unpleasant flavors, and improve the release and uptake of nutraceuticals in the body (149). As a result, nanoencapsulation has progressed from laboratory research to real-world formulation strategies across beverages, dairy products, supplements, snacks, and edible oils, reflecting a broader industry trend. The global market for nanoencapsulation in foods and nutraceuticals has reached multi-billion dollar levels- valued around USD 3.45 billion in 2024- and is projected to grow rapidly (on the order of 8–10% CAGR) in the coming decade (150). North America and Europe currently lead in adoption (accounting for roughly two-thirds of the market by revenue), while the Asia-Pacific region is the fastest-growing market driven by rising demand for functional foods. These commercial developments and market trends underscore a transition from bench to marketplace, as nanoencapsulated nutraceuticals become increasingly mainstream.

**Table 4:** Commercial applications of nanoencapsulated nutraceuticals in food products.

Category	Common nanoencapsulated nutraceuticals	Nanoencapsulation techniques used	Functional benefits	Current limitations	Reference
Functional beverages	Fat-soluble vitamins (e.g., D, E), carotenoids, polyphenols	Nanoemulsions, micelles, colloidal lipid nanocarriers	Enhanced stability, clarity, and absorption; improved taste masking	Thermal and pH instability during processing; high emulsifier requirement; shelf-life concerns	(151, 152)
Dairy products	Vitamins (e.g., D3), omega-3 (EPA/DHA), curcumin, probiotics, polyphenols (e.g., olive extract)	Nanoliposomes, solid lipid nanoparticles, biopolymer nanocarriers	Oxidation prevention, flavor masking, probiotic viability, antioxidant enhancement	Acidic conditions may destabilize nanocarriers; limited heat resistance during pasteurization; interactions with matrix components	(156–160)
Dietary supplements and capsules	Liposomal vitamins (C, D3, B12), minerals (Fe, Ca, Zn), curcumin, CoQ10, glutathione, probiotics, omega-3 oils	Liposomes, nanoemulsions, SLNs, NLCs, polymeric nanoparticles	Strong bioavailability improvements, stability under GI conditions, multi-active delivery	High cost of production; formulation complexity for multicomponent blends; regulatory challenges for nanoform claims	(161–163)
Functional snacks	Omega-3 (EPA/DHA), probiotics, vitamins, minerals, polyphenols (e.g., grape seed, turmeric)	Spray-dried nanoemulsions, starch/lipid matrix nanoparticles, cyclodextrin complexes	Stable delivery without flavor compromise; heat resistance; consumer-accepted formats	Flavor–texture compatibility; probiotic viability in storage; oxidation during baking or extrusion	(167, 168)
Encapsulated oils and lipid nutraceuticals	Omega-3 oils (fish oil, algal DHA), essential oils (e.g., clove, ajwain), fat-soluble vitamins (D, E), carotenoids (astaxanthin)	Nanoemulsions, solid lipid nanoparticles, protein/polysaccharide biopolymer capsules	Reduced rancidity, better solubility, antimicrobial and antioxidant retention	Sensory instability (fishy odor); emulsifier selection affects flavor; oxidation risk under heat/light	(169–184)

### 4.1 Functional beverages

One of the most active areas of commercial uptake is in functional beverages, where nanoencapsulation enables incorporation of insoluble or sensitive nutraceuticals into drinks without sacrificing clarity, taste, or efficacy. Nanoemulsion technology is especially prevalent – extremely fine oil-in-water emulsions (droplet sizes in the tens of nanometers) allow fat-soluble vitamins, carotenoids, cannabinoids, and polyphenols to be stably dispersed in aqueous beverage formulations. Recent market data indicate that nanoemulsions were used in 30–75% of new beverage product launches by the early 2020s, facilitating fortification of drinks with vitamins and plant bioactives that were previously impractical to deliver in liquid form. For example, Nestlé introduced a new range of vitamin-infused beverages in 2023 that utilize nanoencapsulation to improve nutrient delivery; this product line sold over 20 million units within 9 months of launch and expanded to 27 countries. While specifics of Nestlé’s formulation are proprietary, it likely employs lipid-based nanocarriers for fat-soluble vitamins (e.g., vitamins D or E) to enhance stability and absorption. Similarly, numerous start-ups and beverage brands have released “nanoboosted” functional drinks- in 2023 alone, over 70 beverage products across North America and Asia-Pacific used nanoemulsion technology to fortify drinks with ingredients such as curcumin, coenzyme Q10, and botanical extracts (148). Companies have introduced nano-formulated drinks to fortify beverages without compromising clarity or flavor. For instance, Jamba Juice (United States) developed a smoothie additive called *“Daily Boost”* that contains nanoencapsulated vitamins and botanical bioactives, allowing easy incorporation of these nutrients into drinks (151). Ingredient manufacturers have also leveraged nanoemulsions for beverages; Wild Flavors Inc. created a stable “Color Emulsion” based on carotenoid nanoparticles to provide natural colors (beta-carotene, apo-carotenal, paprika extract) in clear drinks without turbidity (152). These nanoemulsion systems improve the dispersibility and stability of otherwise insoluble additives in beverage products, and have been shown to increase the bioavailability of vitamins in drinks compared to conventional formulations (151). Overall, the beverage industry’s adoption of nanoencapsulation has enabled new product formulations (e.g., clear vitamin-fortified drinks) and improved the functional performance of drinks (enhanced nutrient delivery, stability, and taste masking), which is a significant commercial milestone for nutraceutical.

### 4.2 Dairy and fermented products

The dairy industry (including dairy alternatives) has also embraced nanoencapsulation to develop value-added functional products. Milk, yogurt, cheeses, and plant-based dairy analogs are being fortified with nanoencapsulated vitamins, minerals, and phytochemicals to improve nutritional profiles and shelf stability (153–155). A key driver in this sector is the need to protect and uniformly distribute sensitive micronutrients in complex food matrices. Vitamin D_3_, for example, is poorly soluble and degrades during storage; several dairy companies and researchers have addressed this by encapsulating vitamin D in lipid-based nanoparticles for fortification of milk and yogurt (156). Likewise, omega-3 fatty acids from fish oil have been nanoencapsulated and added to dairy foods to enrich them with DHA/EPA without causing oxidation or off-flavors. A recent formulation encapsulated skipjack tuna oil into nanoliposomes (via ethanol injection technique) and successfully blended these into pasteurized cow’s milk as a functional omega-3 fortificant (156). The nano-lipid carriers prevented omega-3 oxidation, preserving DHA/EPA stability in the milk. Commercial infant and toddler nutrition products have similarly utilized micro/nano-encapsulated fish oils (or algal oils) to deliver DHA in milk powder formulas, indicating industry confidence in these delivery systems (though such applications often use microencapsulation on the 100–1,000 nm borderline, e.g., spray-dried emulsions). Another example, Gonçalves et al. (155) developed a stirred yogurt enriched with curcumin-loaded SLNs as a prototype functional dairy product. The curcumin-SLN fortification showed no adverse impact on the yogurt’s taste, texture, or fermentation characteristics during 30 days of storage, while successfully imparting the bioactive properties of curcumin to the yogurt (151). This indicates that nanodelivery systems (like SLNs and liposomes) can integrate sensitive nutraceuticals (e.g., curcumin, vitamins, omega-3 fatty acids, probiotics) into yogurt or milk without destabilizing the dairy matrix. Indeed, other research has encapsulated vitamin D and omega-3 oils in biopolymer nanocarriers for addition to milk, achieving improved nutrient stability and bioaccessibility (157). As food technology and regulations advance, we can expect to see nutraceutically fortified dairy products making use of nanoencapsulation to enhance health benefits.

Beyond fortification, nanoencapsulation has been applied to incorporate bioactive plant compounds into fermented dairy for added health benefits. For example, olive leaf extract (rich in polyphenols) has been entrapped in nanoliposomes and introduced into yogurt, which improved the product’s antioxidant activity and consumer acceptability (reducing syneresis and maintaining flavor) in trials (158). Similarly, researchers have developed nanoscale emulsions of clove essential oil- a natural antimicrobial- and integrated them into soft cheese, achieving extended shelf-life and oxidative stability in the cheese without imparting strong flavor (159, 160). These approaches illustrate how biopolymer nanocarriers (e.g., protein or polysaccharide-based nanoparticles) can serve as delivery vehicles for nutraceuticals in dairy matrices, releasing the actives gradually during storage or digestion. Industry uptake of such innovations is underway. According to market analyses, at least 18 new yogurt and cheese products utilizing nanotechnology were launched in 2023, many focusing on texture improvement, probiotic delivery, or extended freshness (148). For instance, AquaNova AG (Germany) recently developed a plant-based nanoemulsion system for dairy-alternative beverages; by 2024 this nano-formulation had been incorporated into 18 new plant-based milk and shake products, capturing about 8% of the dairy-alternative market in Germany. This product (based on AquaNova’s NovaSOL platform) uses self-assembling micelles from natural surfactants to encapsulate fat-soluble nutrients, thereby enriching soy or oat milks with vitamins and omega-3 s in a stable, homogenous way (148). These real-world examples signal growing industry adoption of nanoencapsulation in the dairy sector. Traditional dairy companies and functional food brands are leveraging nanocarriers- including casein micelles, SLNs like Precirol-based particles and polysaccharide nanoparticles- to create fortified milks, yogurts with added probiotics and antioxidants, and cheeses with improved stability. By addressing issues like nutrient instability and distribution at the nanoscale, these products can deliver clinically meaningful doses of nutrients (e.g., vitamin D, omega-3, and polyphenols) in a convenient daily food format. The trend is not limited to Western markets; fortified yogurts and dairy drinks using nanotech have also emerged in Asia (e.g., nanocalcium or vitamin-fortified milk in China) and the Middle East, reflecting a global interest in “nanoceutical” dairy foods.

The examples above illustrate that different nanoencapsulation approaches can achieve similar functional outcomes in dairy matrices. Notably, both liposomal delivery of polyphenols (olive leaf extract in yogurt) and nanoemulsion-based delivery of essential oils (clove oil in cheese) improved product stability and consumer acceptability without adverse flavor or texture impacts. This suggests that whether a nanocarrier is lipid-based (e.g., nanoliposomes, SLNs) or biopolymer-based (protein/polysaccharide nanoparticles), it can be tuned to protect sensitive nutraceuticals during processing and storage while remaining organoleptically invisible. Comparing studies, one finds that the optimal nanocarrier often depends on the ingredient and desired effect – for instance, encapsulated omega-3 oils in milk benefited from solid lipid or polymer particles to prevent oxidation, whereas probiotics in yogurt survived better when enclosed in acid-resistant biopolymer capsules. Despite these differences, all studies report significantly higher stability and bioaccessibility for the encapsulated nutrients versus unencapsulated fortification. Importantly, no contradictions in outcomes were seen regarding sensory quality: in each case, the nano-formulations either had neutral sensory impact or improved the product (e.g., reduced yogurt syneresis and maintained flavor with nanoliposomes). The underlying mechanisms are consistent as well – nanocarriers in dairy foods create micro-scale protective reservoirs that shield actives from the harsh dairy processing conditions and release them gradually during digestion. Minor variations in release profiles between carrier types (e.g., a fast release from emulsified nanodroplets vs. a slower, sustained release from solid nanoparticles) explain why some formulations might slightly delay the nutrient’s bioavailability, but in all cases the total uptake is improved compared to non-encapsulated controls. In summary, across diverse dairy applications the literature aligns: nanoencapsulation, whether via liposomes, protein complexes, or other nanoparticles, consistently enhances nutrient stability and efficacy, and careful selection of carrier type (matching the nutraceutical’s properties and the dairy matrix requirements) allows these benefits to be realized without compromising product quality.

### 4.3 Dietary supplements and nutraceutical capsules

The dietary supplement industry has arguably seen the most prolific commercialization of nutraceutical nanoencapsulation. Dozens of companies now market “nanotechnology-enhanced” supplements, especially in the form of liposomal vitamins, mineral nanoparticles, and herbal extract nanoemulsions. The motivation is clear: conventional supplement ingredients often have limited bioavailability (e.g., <20% absorption for some vitamins and curcumin), whereas nano-formulated versions can dramatically increase uptake (161). Liposomal delivery systems – where an active compound is enclosed in tiny phospholipid vesicles – have become a popular solution. These liposomes protect the nutrient from degradation and facilitate direct fusion with cellular membranes, thereby boosting absorption. As a result, liposomal supplements are transitioning from niche products to a major market segment. A recent industry report estimated the global liposomal supplements market will reach ~$378 million in 2025, with a CAGR of ~7.8% through 2033, driven by consumer demand for higher efficacy formulations (162).

Product examples abound. One early success was *liposomal vitamin C*, often sold in liquid sachet form (e.g., Altrient C by LivOn Labs), which studies have shown achieves higher plasma vitamin C levels than conventional ascorbic acid (161). Following this, many brands now offer liposomal multivitamins, glutathione, coenzyme Q10, and herbal extracts. For instance, Quicksilver Scientific, Cymbiotika, and Thorne are among the specialist companies focusing on nano-formulated vitamins and antioxidants (162). The competitive landscape includes dozens of players- both nutraceutical start-ups and established supplement firms- developing proprietary nanoencapsulation techniques. In mid-2024, Sudeep Nutrition (India) launched a line of 13 liposome-encapsulated nutraceutical ingredients under the brand “Lipoboost,” targeting supplement manufacturers and functional food formulators. This range spans common supplements like vitamin C, vitamin D3, B12, magnesium, iron, zinc, calcium, as well as nutraceuticals like glutathione, curcumin, coenzyme Q10, DHA omega-3 (10%), and even melatonin (161). The availability of such ready-made liposomal ingredients indicates how nanoencapsulation is being scaled for industry use: supplement companies can now source active ingredients that are pre-encapsulated at the nanoscale, rather than developing the nanotech in-house. According to Sudeep, these liposomal versions can raise oral absorption dramatically (e.g., from ~20% to ~90% for vitamin C) and are compatible with various dosage forms including liquids, capsules, powders, and even gummies. The rapid proliferation of liposomal products has led commentators to call liposomal encapsulation “the future of nutrition” in the supplement sector.

Beyond liposomes, other nano-delivery formats are finding their way into supplements. Solid lipid nanoparticles (SLNs) and nanostructured lipid carriers (NLCs), for example, are used to formulate fat-soluble botanicals like curcumin and resveratrol into capsules. These lipid-based nanoparticles solidify the bioactive in a sub-100 nm matrix of edible fats (e.g., beeswax, glyceryl behenate) which can improve stability and control release. Polymer-based nanocarriers [e.g., biodegradable polymers like poly (lactic-co-glycolic acid) or natural polymers like alginate- chitosan complexes] have been applied to probiotics and enzymes, enabling them to survive stomach acid and reach the intestine effectively- a feature now being advertised in certain “next-gen” probiotic supplements. Co-encapsulation formats are also emerging: instead of delivering a single active, companies are exploring nanoparticles that carry multiple synergistic ingredients. A notable example is the pairing of curcumin with piperine or resveratrol in one nanocomplex to amplify bioavailability and therapeutic effect. In 2025, Jupiter Neurosciences (United States) announced Nugevia™ MND, a cognitive health supplement that combines a patented resveratrol nano-formulation (JOTROL™) with NovaSOL^®^ Curcumin, a micellar curcumin known for high bioavailability (163). This co-delivery approach targets multiple neuroprotective pathways and exemplifies how supplement innovators are using nanoencapsulation to co-deliver polyphenols and other actives for enhanced efficacy. Although Nugevia MND is launching in late 2025, it highlights a trend toward multi-ingredient nanoformulations in the supplement market, made possible by the versatility of nanocarriers.

The commercial impact of these developments is significant. It is reported that nearly half of new dietary supplements introduced in 2023 featured nanoencapsulated ingredients to improve absorption or stability. In Europe and Asia-Pacific alone, at least 39 new nano-enabled supplement products were rolled out in the past couple of years. Consumer response appears positive, as awareness grows regarding the importance of bioavailability; liposomal and “nano” supplements often command premium pricing yet enjoy strong demand in categories like immune support, anti-aging, and brain health (148, 162). Overall, the supplement industry’s adoption of nanoencapsulation reflects a pragmatic strategy to differentiate products and deliver clinically effective doses of nutrients. By leveraging nanotechnology, supplement makers have been able to overcome absorption barriers (e.g., for curcumin, quercetin, and fat-soluble vitamins) and substantiate claims of superior efficacy, which in turn is fueling market growth and intense competition in the nano-supplement niche.

### 4.4 Functional snacks

Applying nanoencapsulation in solid foods and snacks is more challenging than in liquids, but recent commercial attempts show promise. Nutrition bars, bakery products, and snacks are being enriched with nanoencapsulated nutraceuticals to create convenient functional foods (164–166). The challenge is to maintain ingredient stability during baking or storage and to avoid sensory issues (e.g., no fishy taste from omega-3s, no bitterness from polyphenols). Nanoencapsulation helps by sequestering the bioactive in a protective matrix, often with a tasteless coating, and by ensuring uniform dispersion in the food matrix. A clear example is the fortification of snack bars with omega-3 fatty acids: Sarika et al. (167) developed a protein-rich granola bar enriched with fish oil microcapsules, where the fish oil was first emulsified with milk proteins and then freeze-dried into ~100 μm capsules. The resulting bar provided meaningful amounts of EPA/DHA (82 mg per 100 g) without any rancidity or “fishy” off-flavor, and remained stable under various storage conditions. Building on such R&D, some companies have launched omega-3 fortified bars and baked goods using refined encapsulation techniques (often nanoscale emulsions converted to powders). These products target consumers who want the benefits of fish oil or other supplements in a convenient snack format. Probiotic-enriched snacks are another area: researchers have encapsulated probiotic bacteria together with prebiotic fibers in biopolymer nanoparticles to create synbiotic snack bars that maintain high probiotic viability through shelf life (168). Although still emerging, a few functional snacks with encapsulated *Bacillus coagulans* or Lactobacillus (marketed as “probiotic granola bites” etc.) have appeared, leveraging such technology.

Market evidence suggests the trend is nascent but growing. In 2023, approximately 28 new snack and beverage products were introduced in North America featuring nanoencapsulated vitamins, probiotics, or other actives, aimed at health-conscious consumers (148). These included items like high-fiber cereal bars with encapsulated vitamins, antioxidant-rich snack mixes with nano-encapsulated botanical extracts, and protein bars containing nano-iron or calcium for added minerals. The nano-delivery systems used in solid foods vary: some utilize spray-dried nanoemulsions (yielding fine encapsulated powders that can be blended into dough or coatings), while others use inclusion complexes (e.g., cyclodextrin inclusion of bioactives to mask taste) or matrix nanoparticles (such as starch or lipid matrices loaded with the nutraceutical). It should be noted that regulatory and consumer acceptance for nanotech in foods can be a hurdle, particularly in snacks directly marketed to general consumers (as opposed to supplements). Companies often avoid the term “nano” on labels, instead highlighting the functional benefit (“with high-absorption nutrients” or “stabilized omega-3”). Nonetheless, the presence of nanoencapsulated ingredients in snacks is growing steadily under the umbrella of “advanced food technology.” As encapsulation techniques improve (yielding heat-stable, cost-effective nanopowders), we can expect more fortified breads, cereals, and bars to incorporate these innovations. Industry collaborations are already underway; for example, major ingredient suppliers are working with snack manufacturers to test encapsulated omega-3 and vitamin blends in products like crackers and breakfast bars. The overarching goal is to create shelf-stable functional snacks that deliver clinically relevant nutrients without compromising taste or texture. Early product launches and prototypes indicate that nanoencapsulation can indeed make this possible- offering a path to “snackable nutraceuticals” in the near future.

### 4.5 Encapsulated oils and lipid nutraceuticals

The encapsulation of bioactive oils- such as omega-3 fish oils, algal DHA, medium-chain triglycerides, and essential oils from herbs- represents a crucial commercial application of nutraceutical nanotechnology. These oils are highly valued for health (e.g., omega-3 for heart and brain health; essential oils as antioxidants or antimicrobials) but are chemically fragile and prone to oxidation, and they often have strong tastes/aromas. Nanoencapsulation offers a solution by packaging these lipophilic liquids into carrier systems that shield them from oxygen, light, and heat, and modulate their release. A variety of nano-delivery mechanisms are used for oils:

*Nanoemulsions:* Oil is dispersed as nanoscopic droplets within an emulsion, typically stabilized by emulsifiers. This approach is widely used for omega-3 fish oil supplements; for example, several brands now sell “nano fish oil” softgels, where the fish oil is pre-emulsified into ultra-fine droplets for faster absorption and reduced fishy burps. The small droplet size in such nanoemulsions markedly improves the oil’s bioavailability and physical stability (149). In some cases, the nanoemulsified oil is further spray-dried into a powder, yielding free-flowing encapsulated oil that can be incorporated into capsules, drink mixes, or baked goods. Food ingredient companies have developed powdered omega-3 blends using this technique, allowing incorporation of DHA/EPA into products like infant formula and nutrition shakes while masking the odor.

*SLNs:* Here the oil (or an oily nutraceutical) is entrapped in a solid fat matrix at the nanoscale. SLNs and their variant, nanostructured lipid carriers, are especially useful for delivering fat-soluble vitamins and coenzymes. A recent study demonstrated co-encapsulation of vitamin D_3_ and omega-3 oil in a beeswax-based SLN, yielding a solid nanoparticle that could protect both nutrients and release them in a simulated gastrointestinal environment (88). The authors noted such dual-loaded nanoparticles could be incorporated into functional foods (like fortified margarine or yogurt) to simultaneously deliver vitamin D and omega-3 in a stable form. This co-encapsulation strategy for oils is on the cutting edge of product development, potentially enabling synergistic combinations in a single ingredient.

*Biopolymer nanocapsules:* Natural polymers (like gelatin, gum arabic, starch, chitosan) can form nanocapsule shells around oil droplets. These have seen commercial use in protecting essential oils that serve as flavorings or natural preservatives. In one application, nanoencapsulated *Trachyspermum ammi* essential oil (in an alginate matrix) was used to coat turkey meat, significantly extending its refrigerated shelf-life by inhibiting Listeria growth (169). Food companies are testing similar nanoencapsulated essential oil systems to replace synthetic additives in meats, dairy, and beverages. Another example is the use of clove oil nanoemulsions in fresh-cut apples and raw minced beef to prevent mold and oxidation in high-fat dairy (170). The encapsulated form prevents the potent oil from impacting flavor while slowly releasing its protective compounds.

Commercially, encapsulated omega-3 oils are among the most successful implementations of this technology. Many fortified foods and supplements touting omega-3 now contain encapsulated forms: e.g., certain milk brands enriched with DHA use microencapsulated fish oil so that the milk remains odorless (149, 171, 172); some bread and pasta products incorporate encapsulated flaxseed oil to increase omega-3 content without affecting baking performance (173–175). On the supplement side, high-absorption omega-3 capsules often employ nanoemulsion gels or enteric-coated nanocapsules to improve uptake (176–178). The inclusion of astaxanthin (a lipid-soluble antioxidant) alongside fish oil in a nanocarrier is another innovation seen in premium omega-3 supplements, aiming to both protect the oil and provide additional health benefits (179). More industry suppliers have developed stabilized omega-3 encapsulates (though typically micro-scale) for food fortification, and now newer entrants are focusing on nanoscale encapsulates for even better performance.

Notably, the industrial adoption of oil nanoencapsulation spans multiple sectors: functional foods (e.g., beverages with turmeric or curcumin oil nano-dispersed for anti-inflammatory effect), cosmeceuticals and nutricosmetics (e.g., skin health supplements with nano-encapsulated borage oil or carotenoids), and feed/animal nutrition (encapsulated oils in livestock feed to improve meat fatty acid profiles) (180, 181). The versatility of lipid nanocarriers and the clear need to stabilize unsaturated oils ensure that this area remains a priority for R&D. Current market analyses project robust growth in encapsulated oils usage; for instance, the demand for nano-encapsulated omega-3 ingredients is growing in Asia as consumers seek fortified staples (150). With encapsulation techniques improving oxidative stability by over 50% and increasing shelf-life, manufacturers can more confidently include omega-3 or essential oils in their products.

Although various nanoencapsulation formats are employed for oils, no single method is universally “best” – each offers distinct advantages, and comparative studies show the ideal choice depends on the application. For example, nanoemulsions excel at fast absorption due to their extremely high surface area, and indeed emulsified *ω*-3 fish oil droplets have shown markedly quicker uptake and higher bioavailability than traditional oil supplements (176, 182–184). However, if not properly stabilized, those same tiny droplets could oxidize more readily; this seemingly contradictory risk is mitigated by formulation strategies such as adding emulsifiers and co-encapsulated antioxidants (e.g., curcumin or resveratrol) to quench free radicals. In contrast, solid lipid nanoparticles (SLNs/NLCs) provide a crystalline fat matrix that gives superior oxidative protection and controlled release – a clear benefit for highly unsaturated oils. Studies like the beeswax SLN co-loading vitamin D₃ and DHA showed that such solid nanocarriers can simultaneously protect multiple actives and release them in a targeted manner. The trade-off is a slightly slower release profile than nanoemulsions, which can actually be advantageous for delivering oils past the stomach. Biopolymer nanocapsules (e.g., chitosan or alginate-coated oil droplets) offer the flexibility of using GRAS food polymers and can double as edible coatings in foods; their efficacy relies on forming a uniform, oxygen-impermeable shell. Comparative data indicate that all these approaches dramatically outperform unencapsulated oils in stability (often reducing oxidation rates by >50%) and *in vivo* effectiveness. Where they differ is in performance nuances: nanoemulsions are ideal for clear beverages and rapid delivery, SLN/NLC systems are preferred for fortifying foods requiring long shelf-life and for combining fat-soluble nutrients, and polymer nanocapsules are useful when a natural additive label is desired (e.g., using gelatin or gum arabic to encapsulate essential oils in meats). Importantly, these methods are not mutually exclusive – manufacturers often choose based on the specific need, sometimes even integrating multiple strategies (for instance, creating a nanoemulsion of fish oil then spray-drying it into a polymer-coated powder). The literature thus shows a convergence in outcome (enhanced stability/bioavailability) despite different techniques, with any divergences largely explained by mechanism: each nanocarrier system leverages a particular physicochemical principle (small droplet size, crystalline fat barrier, or polymer film) to protect and deliver lipids. By analyzing these studies side-by-side, one can conclude that nanoencapsulation reliably resolves the common fragility and taste issues of bioactive oils, and ongoing research is refining which nanoscale approach best amplifies the health benefits of a given oil in a given product context.

## 5 Challenges and prospective

**Figure 4:**
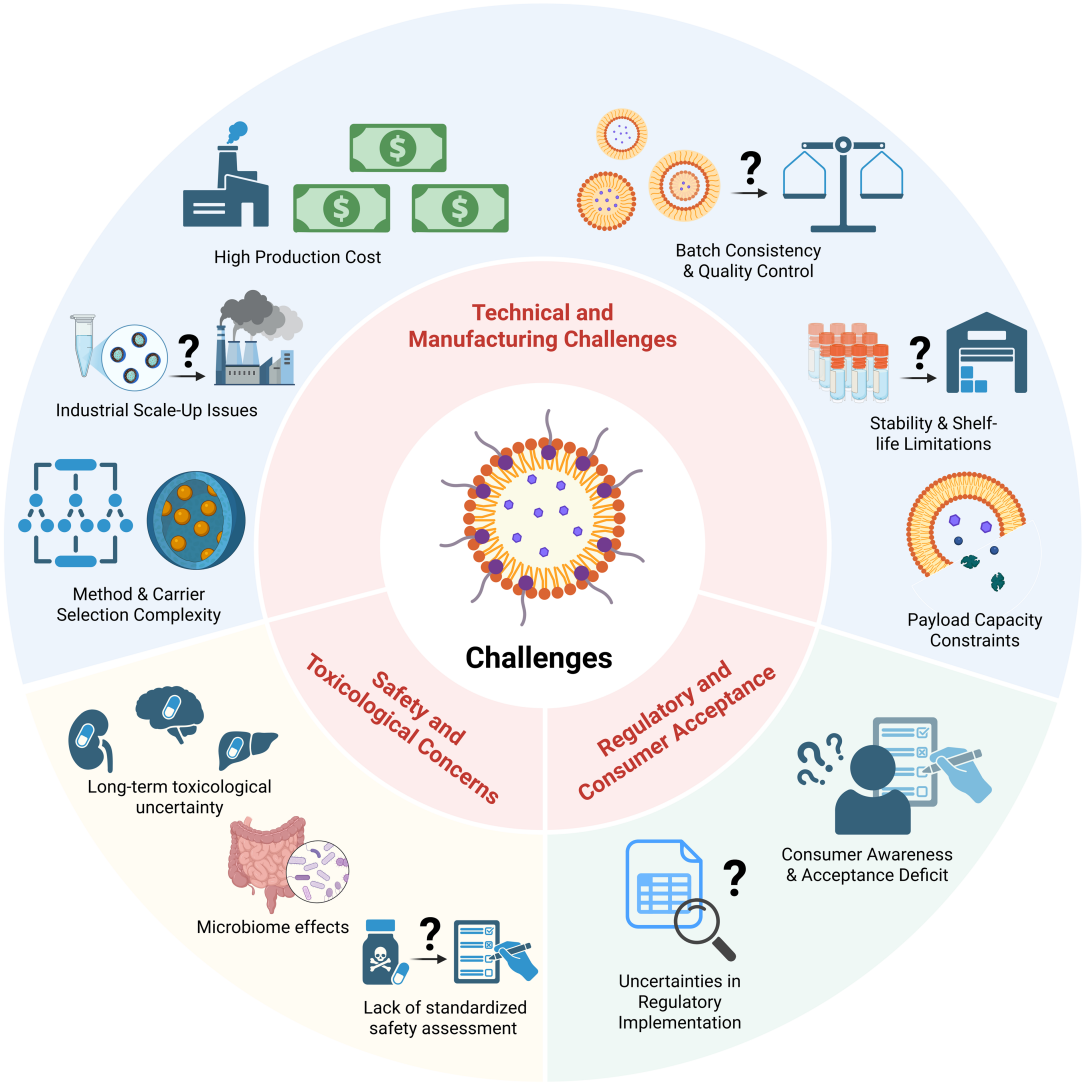
Key challenges in advancing nutraceutical nanoencapsulation technologies. The schematic illustration highlights three interrelated domains of challenges: (i) Technological barriers, including the complexity of selecting optimal encapsulation methods and carriers, difficulties in scaling up production, high manufacturing costs, limited payload capacity, and quality control issues; (ii) Safety concerns, centering on the incomplete understanding of long-term toxicological effects, uncertainties in nanoparticle absorption, distribution, metabolism, and excretion, and potential impacts on the gut microbiome and immune system; and (iii) Regulatory and public acceptance challenges, reflecting the lack of harmonized global regulatory frameworks, uncertainties in regulatory implementation and industrial compliance, and the mixed attitudes of consumers toward nano-enabled foods. Created with BioRender.com.

Despite the notable progress made in the development and application of nanoencapsulation, considerable challenges still need to be addressed before its full potential in nutraceuticals can be realized. These challenges span technical and manufacturing issues, safety and toxicological uncertainties, as well as regulatory and consumer acceptance concerns (Figure 4). Addressing these issues will be crucial for the sustainable translation of nanoencapsulated nutraceuticals from research to mainstream use.

### 5.1 Technical and manufacturing challenges

Developing effective nanoencapsulation systems for nutraceuticals is technically complex. Formulators must determine not only the optimal encapsulation technique but also the most suitable nanocarrier type for each bioactive compound- a nontrivial task given the diverse physicochemical properties of nutraceuticals (185). For instance, a method that works well for a hydrophobic polyphenol might be ill-suited for a water-soluble vitamin, necessitating case-by-case optimization (151, 157).

Moreover, scaling up these nanosystems from the lab to industrial production poses major difficulties. Many nanoencapsulation methods (e.g., high-pressure homogenization, electrospinning, microfluidic assembly) require specialized equipment and precise control, which can be hard to replicate cost-effectively at manufacturing scale (62, 186). The high production costs associated with nanotechnologies, which arise from the use of expensive materials, high energy requirements, and stringent processing conditions, currently limit their broad adoption in the food industry. Ensuring batch-to-batch consistency and quality control of nanoscale particles is another concern, since slight process variations can alter particle size or encapsulation efficiency. Early nano-delivery systems such as liposomes and nanosuspensions initially suffered from storage instability (e.g., payload leakage and particle aggregation), but subsequent formulation optimization has greatly improved their robustness and yielded storage-stable products at scale (187, 188). Researchers have made progress (for example, second-generation lipid carriers and improved emulsifiers help maintain stability), but maintaining nanocarrier integrity during food processing, storage, and distribution remains challenging. Additionally, certain nanocarriers still face payload limitations- while NLCs improved capacity over solid lipid nanoparticles, some highly bioactive nutraceuticals require even higher loading or multiple compound co-entrapment, pushing existing techniques to their limits (189–193). While promising results continue to emerge from academic labs, the industrial maturity of most nanoencapsulation strategies remains limited. Commercial use is still largely confined to nanoemulsions in beverages and liposomal formulations in dietary supplements, which represent relatively high Technology Readiness Levels (TRLs 7–8). In contrast, systems such as microfluidic-assembled nanocapsules or electrosprayed powders remain below TRL 5 due to cost, complexity, or scalability constraints (194, 195). Another challenge lies in the translation of optimized formulations across food matrices: a nanoemulsion stable in an aqueous drink may destabilize in an emulsion-rich dairy product, highlighting the need for matrix-specific revalidation (196). From a cost perspective, nanoencapsulation can increase ingredient costs by 20–50% depending on carrier material and process energy demand, which may not be feasible for commodity products unless consumer-perceived value justifies the premium (197). Addressing these issues calls for clearer TRL benchmarks, standardized food-grade protocols, and collaboration between researchers, food technologists, and regulatory bodies.

In summary, from a technology standpoint, the field must continue improving encapsulation methods that are scalable, cost-effective, and reliable for mass production, ensuring that nano-formulated nutraceuticals can be manufactured consistently and economically without sacrificing performance.

### 5.2 Safety and toxicological concerns

Even as nanoencapsulation solves bioavailability issues, it introduces new questions about the safety of ingesting nanoparticles over time. To date, many nutraceutical nanocarriers are composed of food-grade, biodegradable materials (e.g., lipids, proteins, polysaccharides) and are generally recognized as safe in their conventional (non-nano) form. Nonetheless, when reduced to the nanoscale, these materials may interact with biological systems in novel ways, and our understanding of their long-term toxicological profile is incomplete (198, 199). One major uncertainty is how nanoparticles behave and persist in the human body. Digestible nanocarriers (like protein or lipid-based particles) are expected to break down into absorbable nutrients, but the kinetics of their degradation and absorption are still being characterized (200, 201). There is concern that indigestible or very small nanoparticles could bypass normal digestive processes and accumulate in tissues (202). Studies on inorganic food-grade nanoparticles illustrate potential risks: particles <100 nm can cross the gut epithelium more readily than larger ones and have been found to distribute to the liver, spleen or other organs. Rodent studies using metal oxide and silver nanoparticles have shown that high experimental doses of such materials can induce oxidative stress and inflammatory responses in the liver and gut, although these doses far exceed typical human dietary exposures (203–207). In the context of nutraceuticals, most engineered nanocarriers would be constructed from organic biomolecules rather than metals, which are presumed safer, but the possibility of unexpected biointeractions remains. For example, the high surface reactivity of nanoscale particles could disrupt cellular membranes or protein functions in ways larger particles do not. Another aspect under investigation is the impact of ingested nanoparticles on the gut microbiome. Because some nanoparticles (like chitosan or certain emulsifiers) have antimicrobial properties, they might alter the composition of intestinal flora with long-term use (208, 209). Although no severe adverse health effects of nutraceutical nanocarriers have been reported in humans so far, experts emphasize that chronic exposure studies are lacking (208). Nutraceuticals are often consumed daily over months or years, so even subtle toxicological effects could become significant over time. Yet, performing long-term toxicology studies and dietary intervention trials for each new nano-formulation is resource-intensive, and regulatory agencies have not fully standardized the requirements. Indeed, a 2022 analysis noted that there is still no globally consistent methodology for safety assessment of nano-enabled foods and nutraceuticals, leading to fragmented and case-by-case evaluations (210). This uncertainty creates a “precautionary gap”- both regulators and manufacturers may be cautious, delaying product approvals until more safety data are available. In essence, while nanoencapsulation holds nutritional promise, a parallel effort is needed to rigorously evaluate and ensure the health safety of these novel delivery systems. This includes elucidating their absorption, distribution, metabolism, and excretion pathways, and verifying that they do not induce toxicity at intended doses. The good news is that preliminary studies on many biopolymer- or lipid-based nanocarriers suggest minimal acute toxicity, as they are often metabolized like normal nutrients (208). Nevertheless, the field is still grappling with questions about subtle or rare effects- for instance, whether nanoparticles might trigger unexpected immune responses or cross the blood–brain barrier and elicit neuroeffects (211–213). Ongoing research is focusing on these issues, and it is widely agreed that additional *in vivo* investigations, particularly studies on chronic exposure and reproductive toxicity, are required to confidently establish comprehensive safety profiles (209, 214). Until such data are amassed and standardized testing protocols are in place, safety will remain a pivotal concern in the advancement of nano-nutraceutical products (208, 211).

### 5.3 Regulatory and consumer acceptance challenges

Hand-in-hand with safety concerns are the regulatory and public acceptance challenges for nanoencapsulated nutraceuticals. Regulatory agencies around the world are aware of nanotechnology’s rise in foods and supplements, but legislation has lagged behind the science. At present, there is no universal regulatory framework specifically tailored to nanoencapsulated food ingredients- most countries lack explicit rules or standards for evaluating and approving nanotech in the food sector (215–217). In the European Union, any “engineered nanomaterial” added to food is treated as a novel food additive and requires pre-market authorization, including thorough risk assessment, and must be labeled as “nano” on the ingredient list (218). The European Food Safety Authority (EFSA) has updated its guidance (in 2021) to outline a tiered risk assessment approach for nanomaterials in food/feed, covering key areas like particle characterization, exposure estimation, toxicological testing, and genotoxicity evaluation (219–222). This guidance reflects a more precautionary stance and provides industry with a clearer roadmap on the data needed for nano-scale ingredients. Similarly, the U. S. FDA has issued guidances (most recently in 2022) for nanotechnology in food and cosmetics, recommending that manufacturers consult the FDA early and conduct comprehensive safety tests for products involving nanoscale materials. However, these are guidances rather than dedicated regulations; in the U. S., nanoencapsulated nutraceuticals typically fall under existing dietary supplement regulations, with an expectation that companies ensure safety and label accuracy but no nano-specific labeling requirement (223–226). Other regions (Canada, Asia-Pacific countries, etc.) are gradually formulating their own approaches, often drawing on EU or U. S. principles (227–229). The lack of harmonized international standards means a company faces a patchwork of requirements: a nano-formulated vitamin might be freely sold in one country but considered novel in another, requiring extensive approval processes. This regulatory uncertainty can stifle innovation and complicate global marketing of nano-nutraceuticals. Equally important is the perception of consumers. Public attitudes toward “nanofood” have been mixed, often colored by limited understanding of nanotech and memories of past food safety controversies (230–233). On one hand, if consumers are clearly informed of the health benefits (e.g., “this product uses nanotechnology to improve vitamin absorption”) and assured of safety, many indicate openness to such products, especially for clear functional benefits like disease prevention. On the other hand, the idea of unseen nanoparticles in food raises “fear of the unknown” in some people, and negative media coverage could amplify such fears. Studies have found that using terms like “nanoencapsulation” can trigger more concern than terms like “emulsion” or “colloid,” even if scientifically they refer to similar structures. Thus, transparent risk–benefit communication is vital to avoid consumer backlash (230–233). From a broader perspective, regulators and industry share the goal of protecting consumers and maintaining public confidence in the food supply. High-profile missteps (e.g., if a nano-supplement were implicated in an illness) could set back the field substantially. This makes it all the more crucial to have stringent safety evaluation and oversight in place- as a means to prevent harm and to demonstrate to the public that nano-enabled foods are responsibly managed. In summary, while progress has been made through the efforts of key bodies such as EFSA, USFDA, and WHO in drafting comprehensive guidelines and risk assessment frameworks for food nanomaterials, navigating the regulatory landscape for nanoencapsulated nutraceuticals continues to be challenging, and the absence of uniform, mandatory global regulations must be addressed for these technologies to gain widespread acceptance. In tandem, consumer education efforts will be needed to dispel misconceptions and highlight the tangible health benefits that nanotechnology can provide in nutrition, so that the public embraces rather than fears these innovations.

Taken together, these toxicological, regulatory, and perception-related dimensions are deeply interlinked. Regulatory hesitancy often stems from limited long-term safety data, while consumer acceptance depends not only on scientific assurance but also on transparent communication and regulatory credibility. As such, advancing nano-nutraceutical adoption will require a coordinated effort across scientific validation, risk regulation, and public engagement.

### 5.4 Future prospects

Looking ahead, concerted efforts by scientists, industry, and regulators are expected to mitigate the above challenges and unlock new frontiers for nanoencapsulation in nutrition. A priority is to clarify how nanocarriers behave in biological and environmental contexts and to translate those insights into safer, smarter designs (208, 234, 235). For example, researchers are increasingly focusing on biodegradable and biocompatible nanocarriers that can deliver nutraceuticals and then harmlessly degrade into metabolizable by-products. Future work should move beyond qualitative claims of “biodegradability” toward quantitative degradation kinetics and body-burden profiles in human-relevant models, so that nanocarriers can be compared on the basis of standardized “end-of-life” fingerprints across organs and time scales. Coupling such fate mapping with life-cycle assessment would allow nanoencapsulation platforms to be screened not only for efficacy and safety, but also for climate and resource footprints, enabling rational “safety- and sustainability-by-design” strategies. Using naturally derived polymers (e.g., modified starches, chitosan, alginate, and gelatin) and lipids not only leverages their GRAS status but also minimizes the risk of long-term persistence of nanoparticles in tissues or ecosystems (236–238). Emerging “green” nanotechnologies even explore edible nanocarriers that come from food itself- for instance, plant-derived exosome-like vesicles have been proposed as natural nanocapsules for dietary bioactives, offering innate biocompatibility and low immunogenicity (239–241). Such bio-inspired approaches could address safety and regulatory concerns by blurring the line between natural food structures and engineered delivery systems and by embedding nanoencapsulation into a circular bioeconomy that valorizes agricultural side streams as carrier materials.

Alongside materials innovation, advances in analytical and computational tools will play a key role in moving the field forward. High-resolution characterization techniques (for size, surface properties, etc.) and *in vitro* gut models are being refined to better predict how nanoencapsulated nutrients behave during digestion and absorption (242–245). In particular, gut-on-chip and multi-organ-on-chip platforms are beginning to recreate key mechanical and biochemical cues of the intestine and its crosstalk with liver or immune tissues, providing a controllable setting to study how particle size, interfacial structure and release profiles shape absorption, barrier function and immunomodulation of nutraceuticals (246). These microphysiological systems can be linked with in vitro digestion data and omics-level readouts to generate rich training sets for computational models. Complementary *in silico* modeling and AI can integrate these multi-scale data to predict nanocarrier behavior, identify potential hotspots of accumulation or toxicity, and support safer-by-design optimization (247–253). In the context of regulation, such models could be aligned with New Approach Methodologies (NAMs) and emerging qualification frameworks for nanomaterial risk assessment, providing transparent, reproducible decision rules for when nanoencapsulated nutraceuticals are sufficiently characterized for market entry (254). Over time, linking organ-on-chip data, *in silico* digestion models and AI-based hazard prediction into a single “safety-by-design” pipeline would move nanoencapsulation from case-by-case evaluations toward more systematic, evidence-based development.

Another promising avenue is the idea of precision nutrition through nanotechnology. In the future, nanoencapsulation might enable targeted and even personalized delivery of nutrients. Concepts such as “virtual digital twins” for nutrition envision a computational replica of an individual that integrates genetic, metabolomic, immune, microbiome and behavioral data to simulate the impact of dietary interventions *in silico* (254). When combined with nanoencapsulation, such digital twins could be used to test how different carrier designs, doses and dosing schedules affect simulated post-prandial responses, nutrient status or inflammatory markers before any intervention is implemented *in vivo* (255). Early clinical and real-world studies already indicate that digital twin-enabled personalized nutrition can improve glycemic control, liver fat and cardiometabolic risk in people with type 2 diabetes (256). Integrating nanoencapsulated formulations into these platforms would allow encapsulation strategies to be optimized not only for average bioavailability, but for individual response patterns and co-morbidities. For instance, pH-responsive nanocarriers could release probiotics specifically in the colon for individuals with gut disorders, or iron nanoparticles could be engineered to release only under conditions of anemia (257–259). The integration of wearable health monitors and nano-delivery systems has been envisioned: one could imagine a scenario where a wearable sensor detects a drop in a biomarker (say blood glucose or an inflammatory marker) and triggers the release of a nutraceutical from an ingested nanocarrier, achieving real-time adaptive nutrition (260–264). Concepts such as stimuli-responsive nanocarriers, organ-on-chip–guided formulation screening and digital twin–assisted personalization have been demonstrated at the experimental or pilot level but not yet at industrial scale; taken together, they underscore the potential of nanotechnology to transform nutrient delivery in line with personalized medicine.

Moving forward, the field will need to balance technological ambition with responsible innovation. From a research perspective, at least three medium-term priorities appear particularly important. First, establishing interoperable data infrastructures that connect characterization, digestion, organ-on-chip, *in vivo* and clinical datasets for nanoencapsulated nutraceuticals would enable robust AI models and digital twins, and avoid repeated underpowered studies on isolated endpoints. Second, designing multi-component delivery systems that explicitly account for interactions between carriers, food matrices and the gut ecosystem – including microbiome composition and host genetics – will be essential for translating precision-nutrition concepts into formulations that perform reliably across heterogeneous populations. Third, close collaboration with regulators, ethicists and social scientists is required to co-develop guidance for labeling, risk communication, data protection and equitable access, so that advanced nanoencapsulated products do not only target affluent consumers but address population-level nutrition challenges. Systematic, long-term studies that track the real-world effectiveness and safety of nanoencapsulated nutraceuticals in diverse populations will be essential to move from laboratory demonstrations to trustworthy products on supermarket shelves. Finally, greater attention to socio-economic and ethical dimensions – including consumer acceptance, communication of risk and benefit, labeling and the potential for nanoencapsulation to either widen or reduce nutrition inequities – will determine whether these technologies contribute meaningfully to public health rather than remaining niche solutions.

## References

[1] Bazana MT Codevilla CF de Menezes CR. Nanoencapsulation of bioactive compounds: challenges and perspectives. Curr Opin Food Sci. (2019) 26:47–56. doi: 10.1016/j.cofs.2019.03.005

[2] Dissanayake T Bandara N. Protein-based encapsulation systems for codelivery of bioactive compounds: recent studies and potential applications. Curr Opin Food Sci. (2024) 57:101181. doi: 10.1016/j.cofs.2024.101181

[3] Trujillo J Chirino YI Molina-Jijón E Andérica-Romero AC Tapia E Pedraza-Chaverrí J. Renoprotective effect of the antioxidant curcumin: recent findings. Redox Biol. (2013) 1:448–56. doi: 10.1016/j.redox.2013.09.003, 24191240 PMC3814973

[4] Kaur K Al-Khazaleh AK Bhuyan DJ Li F Li CG. A review of recent curcumin analogues and their antioxidant, anti-inflammatory, and anticancer activities. Antioxidants. (2024) 13:1092. doi: 10.3390/antiox13091092, 39334750 PMC11428508

[5] Constantinescu T Mihis AG. Resveratrol as a privileged molecule with antioxidant activity. Food Chem Adv. (2023) 3:100539. doi: 10.1016/j.focha.2023.100539

[6] Du Q Zhou L Li M Lyu F Liu J Ding Y. Omega-3 polyunsaturated fatty acid encapsulation system: physical and oxidative stability, and medical applications. Food Front. (2022) 3:239–55. doi: 10.1002/fft2.134

[7] Blaner WS Shmarakov IO Traber MG. Vitamin a and vitamin E: will the real antioxidant please stand up? Annu Rev Nutr. (2021) 41:105–31. doi: 10.1146/annurev-nutr-082018-124228 34115520

[8] Bucciantini M Leri M Nardiello P Casamenti F Stefani M. Olive polyphenols: antioxidant and anti-inflammatory properties. Antioxidants. (2021) 10:1044. doi: 10.3390/antiox10071044, 34209636 PMC8300823

[9] Rosales TKO Fabi JP. Pectin-based nanoencapsulation strategy to improve the bioavailability of bioactive compounds. Int J Biol Macromol. (2023) 229:11–21. doi: 10.1016/j.ijbiomac.2022.12.292, 36586647

[10] Anal AK Boonlao N Ruktanonchai UR. Emulsion systems stabilized with biopolymers to enhance oral bioaccessibility and bioavailability of lipophilic bioactive compounds. Curr Opin Food Sci. (2023) 50:101001. doi: 10.1016/j.cofs.2023.101001

[11] Lee MH Do Kim H Jang YJ. Delivery systems designed to enhance stability and suitability of lipophilic bioactive compounds in food processing: a review. Food Chem. (2024) 437:137910. doi: 10.1016/j.foodchem.2023.137910 37931451

[12] Gonçalves A Estevinho BN Rocha F eds. Methodologies for simulation of gastrointestinal digestion of different controlled delivery systems and further uptake of encapsulated bioactive compounds. Trends Food Sci Technol. (2021) 114:510–20. doi: 10.1016/j.tifs.2021.06.007

[13] Yang C Yao L Zhang L. Silk sericin-based biomaterials shine in food and pharmaceutical industries. Smart Mater Med. (2023) 4:447–59. doi: 10.1016/j.smaim.2023.01.003

[14] Yun P Devahastin S Chiewchan N. Microstructures of encapsulates and their relations with encapsulation efficiency and controlled release of bioactive constituents: a review. Compr Rev Food Sci Food Saf. (2021) 20:1768–99. doi: 10.1111/1541-4337.12701, 33527760

[15] Zhou H Zheng B McClements DJ. In vitro gastrointestinal stability of lipophilic polyphenols is dependent on their oil–water partitioning in emulsions: studies on curcumin, resveratrol, and quercetin. J Agric Food Chem. (2021) 69:3340–50. doi: 10.1021/acs.jafc.0c07578, 33689331

[16] Tang C-H. Assembly of food proteins for nano-encapsulation and delivery of nutraceuticals (a mini-review). Food Hydrocoll. (2021) 117:106710. doi: 10.1016/j.foodhyd.2021.106710

[17] Centurion F Basit AW Liu J Gaisford S Rahim MA Kalantar-Zadeh K. Nanoencapsulation for probiotic delivery. ACS Nano. (2021) 15:18653–60. doi: 10.1021/acsnano.1c09951, 34860008

[18] Liu P Li H Li R Geng Y Gong J Xu H . Nanoencapsulation of chitooligosaccharides enhances its oral bioavailability and anti-liver fibrotic effects. Food Res Int. (2022) 157:111471. doi: 10.1016/j.foodres.2022.111471, 35761702

[19] Yang M Ahmad N Hussain M Lu X Xu J Zhong H . A review of recent advances on cyanidin-3-glucoside: the biotransformation, absorption, bioactivity and applications of nano-encapsulation. Food Funct. (2023) 14:6320–45. doi: 10.1039/D2FO03824B, 37403833

[20] Prabhakar P Tripathy S Verma DK Singh S Thakur M Singh AK . Trends and advances in liposome formulation technology with an emphasis on ensuring safety and quality in food and drug applications. Food Biosci. (2025) 69:106913. doi: 10.1016/j.fbio.2025.106913

[21] Tripathy S Verma DK Thakur M Patel AR Srivastav PP Singh S . Encapsulated food products as a strategy to strengthen immunity against COVID-19. Front Nutr. (2021) 8:673174. doi: 10.3389/fnut.2021.673174, 34095193 PMC8175800

[22] Tripathy S Srivastav PP. Sustainable liposomal delivery of *Centella asiatica* polyphenols: β-sitosterol stabilization, LC-MS/MS profiling, and simulated release study. Sustain Food Technol. (2025) 3. doi: 10.1039/D5FB00127G

[23] Feynman RP. B there’s plenty of room at the bottom. Bengaluru: Taylor & Francis eBooks. (1959).

[24] DeFelice SL. The nutraceutical revolution: its impact on food industry R&D. Trends Food Sci Technol. (1995) 6:59–61. doi: 10.1016/S0924-2244(00)88944-X

[25] Allen TM. Liposomes: opportunities in drug delivery. Drugs. (1997) 54:8–14. doi: 10.2165/00003495-199700544-00004, 9361956

[26] Gregoriadis G. Engineering liposomes for drug delivery: progress and problems. Trends Biotechnol. (1995) 13:527–37. doi: 10.1016/S0167-7799(00)89017-4, 8595139

[27] Egbaria K Weiner N. Liposomes as a topical drug delivery system. Adv Drug Deliv Rev. (1990) 5:287–300. doi: 10.1016/0169-409X(90)90021-J

[28] Rudzińska M Grygier A Knight G Kmiecik D. Liposomes as carriers of bioactive compounds in human nutrition. Foods. (2024) 13:1814. doi: 10.3390/foods13121814, 38928757 PMC11202941

[29] Subramani T Ganapathyswamy H. An overview of liposomal nano-encapsulation techniques and its applications in food and nutraceutical. J Food Sci Technol. (2020) 57:3545–55. doi: 10.1007/s13197-020-04360-2, 32903987 PMC7447741

[30] Kirby C Whittle C Rigby N Coxon D Law B. Stabilization of ascorbic acid by microencapsulation in liposomes. Int J Food Sci Technol. (1991) 26:437–49. doi: 10.1111/j.1365-2621.1991.tb01988.x

[31] Gopi S Balakrishnan P. Evaluation and clinical comparison studies on liposomal and non-liposomal ascorbic acid (vitamin C) and their enhanced bioavailability. J Liposome Res. (2021) 31:356–64. doi: 10.1080/08982104.2020.1820521, 32901526

[32] Benech R-O Kheadr E Laridi R Lacroix C Fliss I. Inhibition of *Listeria innocua* in cheddar cheese by addition of nisin Z in liposomes or by in situ production in mixed culture. Appl Environ Microbiol. (2002) 68:3683–90. doi: 10.1128/AEM.68.8.3683-3690.2002, 12147460 PMC124053

[33] Shade CW. Liposomes as advanced delivery systems for nutraceuticals. Integr Med. (2016) 15:33. PMC4818067 27053934

[34] Mirchandani Y Patravale VB. Solid lipid nanoparticles for hydrophilic drugs. J Control Release. (2021) 335:457–64. doi: 10.1016/j.jconrel.2021.05.032, 34048841

[35] Subroto E Andoyo R Indiarto R. Solid lipid nanoparticles: review of the current research on encapsulation and delivery systems for active and antioxidant compounds. Antioxidants. (2023) 12:633. doi: 10.3390/antiox12030633, 36978881 PMC10045442

[36] Hou D Xie C Huang K Zhu C. The production and characteristics of solid lipid nanoparticles (SLNs). Biomaterials. (2003) 24:1781–5. doi: 10.1016/S0142-9612(02)00578-1, 12593960

[37] Mehnert W Mäder K. Solid lipid nanoparticles: production, characterization and applications. Adv Drug Deliv Rev. (2012) 64:83–101. doi: 10.1016/j.addr.2012.09.021 11311991

[38] Kakadia PG Conway BR. Solid lipid nanoparticles: a potential approach for dermal drug delivery. Amer J Pharmacol Sci. (2014) 2:1–7. doi: 10.12691/ajps-2-5A-1

[39] Yu Y Chen D Lee YY Chen N Wang Y Qiu C. Physicochemical and in vitro digestion properties of curcumin-loaded solid lipid nanoparticles with different solid lipids and emulsifiers. Foods. (2023) 12:2045. doi: 10.3390/foods12102045, 37238863 PMC10217647

[40] Gupta T Singh J Kaur S Sandhu S Singh G Kaur IP. Enhancing bioavailability and stability of curcumin using solid lipid nanoparticles (CLEN): a covenant for its effectiveness. Front Bioeng Biotechnol. (2020) 8:879. doi: 10.3389/fbioe.2020.00879, 33178666 PMC7593682

[41] Gokul V Kothapalli P Vasanthan M. A comprehensive review on solid lipid nanoparticles as a carrier for oral absorption of phyto-bioactives. Cureus. (2024) 16. doi: 10.7759/cureus.68339 PMC11444802 39355082

[42] Nguyen T-T-L Duong V-A. Solid Lipid Nanoparticles. Encyclopedia. (2022) 2:952–73. doi: 10.3390/encyclopedia2020063, 35335948

[43] Queiroz MCV Muehlmann LA. Characteristics and preparation of solid lipid nanoparticles and nanostructured lipid carriers. J Nanotheranostics. (2024) 5:188–211. doi: 10.3390/jnt5040012

[44] Jeitler R Glader C König G Kaplan J Tetyczka C Remmelgas J . On the structure, stability, and cell uptake of nanostructured lipid carriers for drug delivery. Mol Pharm. (2024) 21:3674–83. doi: 10.1021/acs.molpharmaceut.4c00392 38838194 PMC11220792

[45] Fathi HA Allam A Elsabahy M Fetih G El-Badry M. Nanostructured lipid carriers for improved oral delivery and prolonged antihyperlipidemic effect of simvastatin. Colloids Surf B Biointerfaces. (2018) 162:236–45. doi: 10.1016/j.colsurfb.2017.11.064 29197789

[46] Shahparast Y Eskandani M Rajaei A Khosroushahi AY. Preparation, physicochemical characterization and oxidative stability of omega-3 fish oil/α-tocopherol-co-loaded nanostructured lipidic carriers. Adv Pharmaceut Bull. (2019) 9:393–400. doi: 10.15171/apb.2019.046, 31592432 PMC6773936

[47] Gonçalves RF Vicente AA Pinheiro AC. Incorporation of curcumin-loaded lipid-based nano delivery systems into food: release behavior in food simulants and a case study of application in a beverage. Food Chem. (2023) 405:134740. doi: 10.1016/j.foodchem.2022.134740 36347204

[48] Walker R Decker EA McClements DJ. Development of food-grade nanoemulsions and emulsions for delivery of omega-3 fatty acids: opportunities and obstacles in the food industry. Food Funct. (2015) 6:41–54. doi: 10.1039/C4FO00723A, 25384961

[49] Walker RM Fish oil nanoemulsions: Optimization of physical and chemical stability for food system applications. Amherst: University of Massachusetts Amherst. (2015).

[50] Aswathanarayan JB Vittal RR. Nanoemulsions and their potential applications in food industry. Front Sustain Food Syst. (2019) 3:95. doi: 10.3389/fsufs.2019.00095

[51] Liu Q Sun Y Cheng J Guo M. Development of whey protein nanoparticles as carriers to deliver soy isoflavones. LWT. (2022) 155:112953. doi: 10.1016/j.lwt.2021.112953

[52] Shakoury N Aliyari MA Salami M Emam-Djomeh Z Vardhanabhuti B Moosavi-Movahedi AA. Encapsulation of propolis extract in whey protein nanoparticles. LWT. (2022) 158:113138. doi: 10.1016/j.lwt.2022.113138

[53] Zou Y Qian Y Rong X Cao K DJ MC Hu K. Encapsulation of quercetin in biopolymer-coated zein nanoparticles: formation, stability, antioxidant capacity, and bioaccessibility. Food Hydrocoll. (2021) 120:106980. doi: 10.1016/j.foodhyd.2021.106980

[54] Reboredo C González-Navarro C Martínez-Oharriz C Martínez-López A Irache J. Preparation and evaluation of PEG-coated zein nanoparticles for oral drug delivery purposes. Int J Pharm. (2021) 597:120287. doi: 10.1016/j.ijpharm.2021.120287, 33524523

[55] Liu M Peng S McClements DJ Chen L Lin S Wang W. Enhancing stability of curcumin-loaded casein nanoparticles by adding liposomal nanoparticles. LWT. (2023) 189:115405. doi: 10.1016/j.lwt.2023.115405

[56] Wang L Jia W Yang Q Cai H Zhao X. Casein nanoparticles as oral delivery carriers for improved bioavailability and hypoglycemic activity of apigenin. Food Hydrocoll. (2024) 146:109194. doi: 10.1016/j.foodhyd.2023.109194

[57] Ma Z Yao J Wang Y Jia J Liu F Liu X. Polysaccharide-based delivery system for curcumin: fabrication and characterization of carboxymethylated corn fiber gum/chitosan biopolymer particles. Food Hydrocoll. (2022) 125:107367. doi: 10.1016/j.foodhyd.2021.107367

[58] Cui H Cheng Q Li C Chen X Lin L. Improving packing performance of lily polysaccharide based edible films via combining with sodium alginate and cold plasma treatment. Int J Biol Macromol. (2022) 206:750–8. doi: 10.1016/j.ijbiomac.2022.02.181 35306012

[59] Lei X Gao B Chen Y Zhao X Qin Y Song Y . A novel polysaccharide-based approach from macromolecular interactions: control of postharvest Botrytis cinerea by mannoprotein and sodium alginate. Food Chem. (2025) 491:145199. doi: 10.1016/j.foodchem.2025.145199, 40587938

[60] Zhang X Wei Z Sun Y Luo T Xue C. Preparation of core–shell hordein/pectin nanoparticles as quercetin delivery matrices: physicochemical properties and colon-specific release analyses. Food Res Int. (2023) 170:112971. doi: 10.1016/j.foodres.2023.112971, 37316013

[61] Huang X Li T Li S. Encapsulation of vitexin-rhamnoside based on zein/pectin nanoparticles improved its stability and bioavailability. Curr Res Food Sci. (2023) 6:100419. doi: 10.1016/j.crfs.2022.100419, 36582445 PMC9792296

[62] Mehta J Pathania K Pawar SV. Recent overview of nanotechnology based approaches for targeted delivery of nutraceuticals. Sustain Food Technol. (2025) 3:947–8. doi: 10.1039/D5FB00122F

[63] Ways TM Lau WM Khutoryanskiy VV. Chitosan and its derivatives for application in mucoadhesive drug delivery systems. Polymers. (2018) 10:267. doi: 10.3390/polym10030267 30966302 PMC6414903

[64] Kim ES Kim DY Lee J-S Lee HG. Mucoadhesive chitosan–gum arabic nanoparticles enhance the absorption and antioxidant activity of quercetin in the intestinal cellular environment. J Agric Food Chem. (2019) 67:8609–16. doi: 10.1021/acs.jafc.9b00008, 31314514

[65] Raghunath I Koland M Saoji SD Bukke SPN Deshpande NS Augustin V . Effects of piperine on intestinal permeation, pharmacodynamics and pharmacokinetics of insulin loaded chitosan coated solid lipid nanoparticles in rats. Sci Rep. (2025) 15:22771. doi: 10.1038/s41598-025-05137-3, 40594447 PMC12218416

[66] Yen Y-W Lee Y-L Yu L-Y Li C-E Shueng P-W Chiu H-C . Fucoidan/chitosan layered PLGA nanoparticles with melatonin loading for inducing intestinal absorption and addressing triple-negative breast cancer progression. Int J Biol Macromol. (2023) 250:126211. doi: 10.1016/j.ijbiomac.2023.126211, 37562466

[67] Rizwan M Yahya R Hassan A Yar M Azzahari AD Selvanathan V . pH sensitive hydrogels in drug delivery: brief history, properties, swelling, and release mechanism, material selection and applications. Polymers. (2017) 9:137. doi: 10.3390/polym9040137, 30970818 PMC6432076

[68] Bachta P Jakhmola V Nainwal N Joshi P Bahuguna R Chaudhary M . A comprehensive study on pH-sensitive nanoparticles for the efficient delivery of drugs. J Adv Biotechnol Exp Ther. (2025) 8:200. doi: 10.5455/jabet.2025.17

[69] Wang J-L Yeh C-H Huang S-H Wu LS-H Chen MC-M. Effects of resistant-starch-encapsulated probiotic cocktail on intestines damaged by 5-fluorouracil. Biomedicine. (2024) 12. doi: 10.3390/biomedicines12081912, 39200376 PMC11351836

[70] Tang H-Y Fang Z Ng K. Dietary fiber-based colon-targeted delivery systems for polyphenols. Trends Food Sci Technol. (2020) 100:333–48. doi: 10.1016/j.tifs.2020.04.028

[71] Feng K Wei Y-s Hu T-g Linhardt RJ Zong M-h Wu H. Colon-targeted delivery systems for nutraceuticals: a review of current vehicles, evaluation methods and future prospects. Trends Food Sci Technol. (2020) 102:203–22. doi: 10.1016/j.tifs.2020.05.019

[72] Wen P Zong M-H Linhardt RJ Feng K Wu H. Electrospinning: a novel nano-encapsulation approach for bioactive compounds. Trends Food Sci Technol. (2017) 70:56–68. doi: 10.1016/j.tifs.2017.10.009

[73] Coelho SC Estevinho BN Rocha F. Encapsulation in food industry with emerging electrohydrodynamic techniques: electrospinning and electrospraying–a review. Food Chem. (2021) 339:127850. doi: 10.1016/j.foodchem.2020.127850, 32861932

[74] Mendes AC Chronakis IS. Electrohydrodynamic encapsulation of probiotics: a review. Food Hydrocoll. (2021) 117:106688. doi: 10.1016/j.foodhyd.2021.106688

[75] Bhushani JA Anandharamakrishnan C. Electrospinning and electrospraying techniques: potential food based applications. Trends Food Sci Technol. (2014) 38:21–33. doi: 10.1016/j.tifs.2014.03.004

[76] Zare M Dziemidowicz K Williams GR Ramakrishna S. Encapsulation of pharmaceutical and nutraceutical active ingredients using electrospinning processes. Nano. (2021) 11:1968. doi: 10.3390/nano11081968, 34443799 PMC8399548

[77] Rüzgar G Birer M Tort S Acartürk F. Studies on improvement of water-solubility of curcumin with electrospun nanofibers. FABAD J Pharm Sci. (2013) 38:143.

[78] Gaydhane MK Sharma CS Majumdar S. Electrospun nanofibres in drug delivery: advances in controlled release strategies. RSC Adv. (2023) 13:7312–28. doi: 10.1039/D2RA06023J, 36891485 PMC9987416

[79] Sun Y Cheng S Lu W Wang Y Zhang P Yao Q. Electrospun fibers and their application in drug controlled release, biological dressings, tissue repair, and enzyme immobilization. RSC Adv. (2019) 9:25712–29. doi: 10.1039/C9RA05012D, 35530076 PMC9070372

[80] Lim L-T Mendes AC Chronakis IS. Electrospinning and electrospraying technologies for food applications. Adv Food Nutr Res. (2019) 88:167–234. doi: 10.1016/bs.afnr.2019.02.005, 31151724

[81] Feng K Huangfu L Liu C Bonfili L Xiang Q Wu H . Electrospinning and electrospraying: emerging techniques for probiotic stabilization and application. Polymers. (2023) 15:2402. doi: 10.3390/polym15102402, 37242977 PMC10224468

[82] Jayaprakash P Maudhuit A Gaiani C Desobry S. Encapsulation of bioactive compounds using competitive emerging techniques: electrospraying, nano spray drying, and electrostatic spray drying. J Food Eng. (2023) 339:111260. doi: 10.1016/j.jfoodeng.2022.111260

[83] Tanhaei A Mohammadi M Hamishehkar H Hamblin MR. Electrospraying as a novel method of particle engineering for drug delivery vehicles. J Control Release. (2021) 330:851–65. doi: 10.1016/j.jconrel.2020.10.059, 33137365

[84] Raval D Kabariya J Hazra T Ramani V. A review on electrospraying technique for encapsulation of nutraceuticals. Int J Chem Stud. (2019) 7:1183–7.

[85] Xu X Tang Q Gao Y Chen S Yu Y Qian H . Recent developments in the fabrication of food microparticles and nanoparticles using microfluidic systems. Crit Rev Food Sci Nutr. (2025) 65:2199–213. doi: 10.1080/10408398.2024.2329967, 38520155

[86] Zhao C Zhu Y Kong B Huang Y Yan D Tan H . Dual-core prebiotic microcapsule encapsulating probiotics for metabolic syndrome. ACS Appl Mater Interfaces. (2020) 12:42586–94. doi: 10.1021/acsami.0c13518, 32869634

[87] Liu K Chen Y-Y Pan L-H Li Q-M Luo J-P Zha X-Q. Co-encapsulation systems for delivery of bioactive ingredients. Food Res Int. (2022) 155:111073. doi: 10.1016/j.foodres.2022.111073, 35400451

[88] Shakeri M Ghobadi R Sohrabvandi S Khanniri E Mollakhalili-Meybodi N. Co-encapsulation of omega-3 and vitamin D3 in beeswax solid lipid nanoparticles to evaluate physicochemical and in vitro release properties. Front Nutr. (2024) 11:1323067. doi: 10.3389/fnut.2024.1323067, 38633604 PMC11021770

[89] Wen C Tang J Cao L Fan M Lin X Liu G . Strategic approaches for co-encapsulation of bioactive compounds: technological advances and mechanistic insight. Foods. (2025) 14:2024. doi: 10.3390/foods14122024, 40565632 PMC12191517

[90] Oanh HT Thu NTH Van Hanh N Hoang MH Hien HTM. Co-encapsulated astaxanthin and kaempferol nanoparticles: fabrication, characterization, and their potential synergistic effects on treating non-alcoholic fatty liver disease. RSC Adv. (2023) 13:35127–36. doi: 10.1039/D3RA06537E 38046630 PMC10691322

[91] Wei Y Wang C Liu X Mackie A Zhang M Dai L . Co-encapsulation of curcumin and β-carotene in Pickering emulsions stabilized by complex nanoparticles: effects of microfluidization and thermal treatment. Food Hydrocoll. (2022) 122:107064. doi: 10.1016/j.foodhyd.2021.107064

[92] Zhan S He M Wu Y Ouyang J. Improved light and ultraviolet stability of curcumin encapsulated in emulsion gels prepared with corn starch, OSA-starch and whey protein isolate. Food Chem. (2024) 446:138803. doi: 10.1016/j.foodchem.2024.138803, 38412810

[93] Drosou C Krokida M. A comparative study of encapsulation of β-carotene via spray-drying and freeze-drying techniques using pullulan and whey protein isolate as wall material. Foods. (2024) 13:1933. doi: 10.3390/foods13121933, 38928875 PMC11203211

[94] Feng Y Li C Jin H Sun Y Jiang H Li Y . Pickering emulsions stabilized by a naturally derived one-dimensional all-in-one hybrid nanostructure. Langmuir. (2025) 41:4748–55. doi: 10.1021/acs.langmuir.4c04712, 39936401 PMC11866913

[95] Barra PA Márquez K Gil-Castell O Mujica J Ribes-Greus A Faccini M. Spray-drying performance and thermal stability of L-ascorbic acid microencapsulated with sodium alginate and gum Arabic. Molecules. (2019) 24:2872. doi: 10.3390/molecules24162872, 31394884 PMC6721127

[96] Farias MD Albuquerque PB Soares PA de Sá DM Vicente AA Carneiro-da-Cunha MG. Xyloglucan from *Hymenaea courbaril* var. courbaril seeds as encapsulating agent of L-ascorbic acid. Int J Biol Macromol. (2018) 107:1559–66. doi: 10.1016/j.ijbiomac.2017.10.016, 28987799

[97] Lim AS Roos YH. Spray drying of high hydrophilic solids emulsions with layered interface and trehalose-maltodextrin as glass formers for carotenoids stabilization. J Food Eng. (2016) 171:174–84. doi: 10.1016/j.jfoodeng.2015.10.026

[98] Sansone F Esposito T Mencherini T Del Prete F Cannoniere AL Aquino RP. Exploring microencapsulation potential: multicomponent spray dried delivery systems for improvement of *Chlorella vulgaris* extract preservation and solubility. Powder Technol. (2023) 429:118882. doi: 10.1016/j.powtec.2023.118882

[99] Rahmani-Manglano NE Fallahasghari EZ Mendes AC Andersen ML Guadix EM Chronakis IS . Oxidative stability of fish oil-loaded nanocapsules produced by electrospraying using kafirin or zein proteins as wall materials. Antioxidants. (2024) 13:1145. doi: 10.3390/antiox13091145, 39334804 PMC11428463

[100] Selim KA Alharthi SS Abu El-Hassan AM Elneairy NA Rabee LA Abdel-Razek AG. The effect of wall material type on the encapsulation efficiency and oxidative stability of fish oils. Molecules. (2021) 26:6109. doi: 10.3390/molecules26206109, 34684694 PMC8538360

[101] Xiao S Ahn DU. Co-encapsulation of fish oil with essential oils and lutein/curcumin to increase the oxidative stability of fish oil powder. Food Chem. (2023) 410:135465. doi: 10.1016/j.foodchem.2023.135465, 36641907

[102] Mishra V Kaur D Singh S Singh DP Krishania M. Sustainable nanofiber synthesis from corn protein meal for enhanced vitamin E and curcumin nutrient delivery in food systems. Sustain Food Technol. (2024) 2:1011–21. doi: 10.1039/D3FB00236E

[103] Kong F Kang S Zhang J Jiang L Liu Y Yang M . The non-covalent interactions between whey protein and various food functional ingredients. Food Chem. (2022) 394:133455. doi: 10.1016/j.foodchem.2022.133455, 35732088

[104] Kalantary S Golbabaei F Latifi M Shokrgozar MA Yaseri M. Feasibility of using vitamin E-loaded poly (ε-caprolactone)/gelatin nanofibrous mat to prevent oxidative stress in skin. J Nanosci Nanotechnol. (2020) 20:3554–62. doi: 10.1166/jnn.2020.17486, 31748051

[105] Ghosh S Sarkar T Das A Chakraborty R. Natural colorants from plant pigments and their encapsulation: an emerging window for the food industry. LWT. (2022) 153:112527. doi: 10.1016/j.lwt.2021.112527

[106] Cheng Y Liu J Li L Ren J Lu J Luo F. Advances in embedding techniques of anthocyanins: improving stability, bioactivity and bioavailability. Food Chem. (2023) 20:100983. doi: 10.1016/j.fochx.2023.100983, 38144721 PMC10740132

[107] Adefegha SA Ogundare PO Oboh G Esatbeyoglu T Papenbrock J. An overview on the mechanisms of encapsulation of polyphenols used to help fight diabetes mellitus. Discov Food. (2025) 5:185. doi: 10.1007/s44187-025-00455-x

[108] Alishahi A Mirvaghefi A Tehrani M Farahmand H Shojaosadati S Dorkoosh F . Shelf life and delivery enhancement of vitamin C using chitosan nanoparticles. Food Chem. (2011) 126:935–40. doi: 10.1016/j.foodchem.2010.11.086

[109] Telles BdSS BSS Garcia-Rojas EE. Curcumin encapsulation through complex coacervation using carboxymethylated tara gum and lysozyme: methodology, characterization, and incorporation in bread. Cienc Agrotec. (2024) 48:e011824. doi: 10.1590/1413-7054202448011824

[110] Zhang C Chen Z He Y Xian J Luo R Zheng C . Oral colon-targeting core–shell microparticles loading curcumin for enhanced ulcerative colitis alleviating efficacy. Chin Med. (2021) 16:92. doi: 10.1186/s13020-021-00449-8, 34551815 PMC8456585

[111] Chen B Ai C He Y Zheng Y Chen L Teng H. Preparation and structural characterization of chitosan-sodium alginate nanocapsules and their effects on the stability and antioxidant activity of blueberry anthocyanins. Food Chemistry. (2024) 23:101744. doi: 10.1016/j.fochx.2024.101744, 39257493 PMC11385793

[112] Ye H Chen T Huang M Ren G Lei Q Fang W . Exploration of the microstructure and rheological properties of sodium alginate-pectin-whey protein isolate stabilized Β-carotene emulsions: to improve stability and achieve gastrointestinal sustained release. Foods. (2021) 10:1991. doi: 10.3390/foods10091991, 34574098 PMC8465917

[113] Sidlagatta V Chilukuri SVV Devana BR Dasi SD Rangaswamy L. Effect of maltodextrin concentration and inlet air temperature on properties of spray dried powder from reverse osmosis concentrated sweet orange juice. Braz Arch Biol Technol. (2020) 63:e20190538. doi: 10.1590/1678-4324-2020190538

[114] Arebo MA Feyisa JD Tafa KD Satheesh N. Optimization of spray-drying parameter for production of better quality orange fleshed sweet potato (*Ipomoea batatas* L.) powder: selected physiochemical, morphological, and structural properties. Heliyon. (2023) 9. doi: 10.1016/j.heliyon.2023.e13078, 36747935 PMC9898653

[115] Nowak D Jakubczyk E. The freeze-drying of foods—the characteristic of the process course and the effect of its parameters on the physical properties of food materials. Foods. (2020) 9:1488. doi: 10.3390/foods9101488, 33080983 PMC7603155

[116] Abdelwahed W Degobert G Stainmesse S Fessi H. Freeze-drying of nanoparticles: formulation, process and storage considerations. Adv Drug Deliv Rev. (2006) 58:1688–713. doi: 10.1016/j.addr.2006.09.017, 17118485

[117] Anusiya G Jaiganesh R. A review on fabrication methods of nanofibers and a special focus on application of cellulose nanofibers. Carbohydr Polym Technol Appl. (2022) 4:100262. doi: 10.1016/j.carpta.2022.100262

[118] de Abreu FJ de Paula Silva CR Oliveira MFS Norcino LB Campelo PH Botrel DA . Microencapsulation by spray chilling in the food industry: opportunities, challenges, and innovations. Trends Food Sci Technol. (2022) 120:274–87. doi: 10.1016/j.tifs.2021.12.026, 36569414 PMC9759634

[119] Timilsena YP Haque MA Adhikari B. Encapsulation in the food industry: a brief historical overview to recent developments. Food Nutr Sci. (2020) 11:481–508.

[120] Nakagawa K Kamisaki H Suzuki T Sano N. Model-based prediction of the moisture sorption kinetics and humidity-induced collapse for freeze–dried cakes. Chem Eng Sci. (2022) 248:117129. doi: 10.1016/j.ces.2021.117129

[121] Shamsher E Khan RS Davis BM Dine K Luong V Somavarapu S . Nanoparticles enhance solubility and neuroprotective effects of resveratrol in demyelinating disease. Neurotherapeutics. (2023) 20:1138–53. doi: 10.1007/s13311-023-01378-0, 37160530 PMC10457259

[122] Pouton CW. Formulation of poorly water-soluble drugs for oral administration: physicochemical and physiological issues and the lipid formulation classification system. Eur J Pharm Sci. (2006) 29:278–87. doi: 10.1016/j.ejps.2006.04.016, 16815001

[123] Williams HD Trevaskis NL Charman SA Shanker RM Charman WN Pouton CW . Strategies to address low drug solubility in discovery and development. Pharmacol Rev. (2013) 65:315–499. doi: 10.1124/pr.112.005660, 23383426

[124] Mu H Holm R Müllertz A. Lipid-based formulations for oral administration of poorly water-soluble drugs. Int J Pharm. (2013) 453:215–24. doi: 10.1016/j.ijpharm.2013.03.054, 23578826

[125] McClements DJ. Edible nanoemulsions: fabrication, properties, and functional performance. Soft Matter. (2011) 7:2297–316. doi: 10.1039/C0SM00549E

[126] McClements DJ Xiao H. Excipient foods: designing food matrices that improve the oral bioavailability of pharmaceuticals and nutraceuticals. Food Funct. (2014) 5:1320–33. doi: 10.1039/C4FO00100A, 24760211

[127] Gomes A Costa ALR Cardoso DD Nathia-Neves G Meireles MAA Cunha RL. Interactions of β-carotene with WPI/tween 80 mixture and oil phase: effect on the behavior of O/W emulsions during in vitro digestion. Food Chem. (2021) 341:128155. doi: 10.1016/j.foodchem.2020.128155, 33045587

[128] Pool H Mendoza S Xiao H McClements DJ. Encapsulation and release of hydrophobic bioactive components in nanoemulsion-based delivery systems: impact of physical form on quercetin bioaccessibility. Food Funct. (2013) 4:162–74. doi: 10.1039/C2FO30042G, 23172078

[129] Brouwers J Brewster ME Augustijns P. Supersaturating drug delivery systems: the answer to solubility-limited oral bioavailability? J Pharm Sci. (2009) 98:2549–72. doi: 10.1002/jps.21650, 19373886

[130] Yang C Wu T Qi Y Zhang Z. Recent advances in the application of vitamin E TPGS for drug delivery. Theranostics. (2018) 8:464–85. doi: 10.7150/thno.22711, 29290821 PMC5743561

[131] Kaiser M Pereira S Pohl L Ketelhut S Kemper B Gorzelanny C . Chitosan encapsulation modulates the effect of capsaicin on the tight junctions of MDCK cells. Sci Rep. (2015) 5:10048. doi: 10.1038/srep10048 25970096 PMC4429556

[132] Wang Y Mo Y Sun Y Li J An Y Feng N . Intestinal nanoparticle delivery and cellular response: a review of the bidirectional nanoparticle-cell interplay in mucosa based on physiochemical properties. J Nanobiotechnol. (2024) 22:669. doi: 10.1186/s12951-024-02930-6, 39487532 PMC11531169

[133] Dinh L Yan B. Oral drug delivery via intestinal lymphatic transport utilizing lipid-based lyotropic liquid crystals. Liquids. (2023) 3:456–68. doi: 10.3390/liquids3040029, 38711572 PMC11073766

[134] Wang T Luo Y. Biological fate of ingested lipid-based nanoparticles: current understanding and future directions. Nanoscale. (2019) 11:11048–63. doi: 10.1039/C9NR03025E, 31149694

[135] He Y Cheng M Yang R Li H Lu Z Jin Y . Research progress on the mechanism of nanoparticles crossing the intestinal epithelial cell membrane. Pharmaceutics. (2023) 15:1816. doi: 10.3390/pharmaceutics15071816, 37514003 PMC10384977

[136] Griffiths G Gruenberg J Marsh M Wohlmann J Jones AT Parton RG. Nanoparticle entry into cells; the cell biology weak link. Adv Drug Deliv Rev. (2022) 188:114403. doi: 10.1016/j.addr.2022.114403, 35777667

[137] Lozano MV Torrecilla D Torres D Vidal A Domínguez F Alonso MJ. Highly efficient system to deliver taxanes into tumor cells: docetaxel-loaded chitosan oligomer colloidal carriers. Biomacromolecules. (2008) 9:2186–93. doi: 10.1021/bm800298u, 18637687

[138] Guo Y Luo J Tan S Otieno BO Zhang Z. The applications of vitamin E TPGS in drug delivery. Eur J Pharm Sci. (2013) 49:175–86. doi: 10.1016/j.ejps.2013.02.006, 23485439

[139] Zhang Z Lu Y Qi J Wu W. An update on oral drug delivery via intestinal lymphatic transport. Acta Pharm Sin B. (2021) 11:2449–68. doi: 10.1016/j.apsb.2020.12.022, 34522594 PMC8424224

[140] Yáñez JA Wang SW Knemeyer IW Wirth MA Alton KB. Intestinal lymphatic transport for drug delivery. Adv Drug Deliv Rev. (2011) 63:923–42. doi: 10.1016/j.addr.2011.05.019, 21689702 PMC7126116

[141] Trevaskis NL Kaminskas LM Porter CJ. From sewer to saviour—targeting the lymphatic system to promote drug exposure and activity. Nat Rev Drug Discov. (2015) 14:781–803. doi: 10.1038/nrd4608, 26471369

[142] Trevaskis NL Charman WN Porter CJ. Lipid-based delivery systems and intestinal lymphatic drug transport: a mechanistic update. Adv Drug Deliv Rev. (2008) 60:702–16. doi: 10.1016/j.addr.2007.09.007, 18155316 PMC7103284

[143] Subramanian P. Lipid-based nanocarrier system for the effective delivery of nutraceuticals. Molecules. (2021) 26:5510. doi: 10.3390/molecules26185510, 34576981 PMC8468612

[144] Chi H Zhang X Chen Z Chen Q Yang B Deng H . Lymph-targeted delivery of CUR-NLCs enhances Oral bioavailability: evidence from a double-catheterized rat model. Pharmaceutics. (2025) 17:1484. doi: 10.3390/pharmaceutics17111484, 41304820 PMC12656303

[145] Sabu C Raghav D Jijith U Mufeedha P Naseef P Rathinasamy K . Bioinspired oral insulin delivery system using yeast microcapsules. Mater Sci Eng C. (2019) 103:109753. doi: 10.1016/j.msec.2019.109753, 31349477

[146] Ma Y He H Xia F Li Y Lu Y Chen D . In vivo fate of lipid-silybin conjugate nanoparticles: implications on enhanced oral bioavailability. Nanomed Nanotechnol Biol Med. (2017) 13:2643–54. doi: 10.1016/j.nano.2017.07.014, 28778838

[147] Kanaya T Williams IR Ohno H. Intestinal M cells: tireless samplers of enteric microbiota. Traffic. (2020) 21:34–44. doi: 10.1111/tra.12707, 31647148

[148] Food nanotechnology market size, share, growth, and industry analysis, by type (nanoparticles, Nanoemulsions, nanosensors), by application (food industry, packaging, nutraceuticals), regional insights and forecast to 2033. (2025). 2025-08-25. Available online at: https://www.marketgrowthreports.com/market-reports/food-nanotechnology-market-114352 (Accessed August 25, 2025).

[149] Venugopalan VK Gopakumar LR Kumaran AK Chatterjee NS Soman V Peeralil S . Encapsulation and protection of omega-3-rich fish oils using food-grade delivery systems. Foods. (2021) 10:1566. doi: 10.3390/foods10071566, 34359436 PMC8305697

[150] Global Nanoencapsulation for food products market size by application (functional foods, snacks and beverages), by type of encapsulated material (proteins, carbohydrates), by encapsulation technology (spray drying, Coacervation), by form of encapsulated product (powder, liquid), by functionality (improved shelf life, enhanced nutrient delivery), by geographic scope and forecast. (2025). Available online at: https://www.verifiedmarketreports.com/product/nanoencapsulation-for-food-products-market-size-and-forecast/.

[151] Nejatian M Darabzadeh N Bodbodak S Saberian H Rafiee Z Kharazmi MS . Practical application of nanoencapsulated nutraceuticals in real food products; a systematic review. Adv Colloid Interf Sci. (2022) 305:102690. doi: 10.1016/j.cis.2022.102690, 35525089

[152] McClements DJ Rao J. Food-grade nanoemulsions: formulation, fabrication, properties, performance, biological fate, and potential toxicity. Crit Rev Food Sci Nutr. (2011) 51:285–330. doi: 10.1080/10408398.2011.559558, 21432697

[153] Almasi K Esnaashari SS Khosravani M Adabi M. Yogurt fortified with omega-3 using nanoemulsion containing flaxseed oil: investigation of physicochemical properties. Food Sci Nutr. (2021) 9:6186–93. doi: 10.1002/fsn3.2571, 34760249 PMC8565221

[154] Matos R Santos Pansarim A Carvalho Brito-Oliveira T Andrade Chaves M Pinho SC. Nanoencapsulation of vitamin D3 by the emulsion inversion point method for enrichment of coconut yogurts. ACS Food Sci Technol. (2022) 2:1899–910. doi: 10.1021/acsfoodscitech.2c00276

[155] Gonçalves RF Rodrigues R Vicente AA Pinheiro AC. Incorporation of solid lipid nanoparticles into stirred yogurt: effects in physicochemical and rheological properties during shelf-life. Nano. (2022) 13:93. doi: 10.3390/nano13010093, 36616003 PMC9823338

[156] Taouzinet L Djaoudene O Fatmi S Bouiche C Amrane-Abider M Bougherra H . Trends of nanoencapsulation strategy for natural compounds in the food industry. PRO. (2023) 11:1459. doi: 10.3390/pr11051459

[157] Akhavan S Assadpour E Katouzian I Jafari SM. Lipid nano scale cargos for the protection and delivery of food bioactive ingredients and nutraceuticals. Trends Food Sci Technol. (2018) 74:132–46. doi: 10.1016/j.tifs.2018.02.001

[158] Tavakoli H Hosseini O Jafari SM Katouzian I. Evaluation of physicochemical and antioxidant properties of yogurt enriched by olive leaf phenolics within nanoliposomes. J Agric Food Chem. (2018) 66:9231–40. doi: 10.1021/acs.jafc.8b02759, 30110548

[159] Hua L Deng J Wang Z Wang Y Chen B Ma Y . Improving the functionality of chitosan-based packaging films by crosslinking with nanoencapsulated clove essential oil. Int J Biol Macromol. (2021) 192:627–34. doi: 10.1016/j.ijbiomac.2021.09.197, 34626727

[160] Ahmadabadi LR Hosseini SE Seyedein Ardebili SM Mousavi Khaneghah A. Application of clove essential oil-loaded nanoemulsions in coating of chicken fillets. J Food Meas Charact. (2022) 16:819–28. doi: 10.1007/s11694-021-01207-y

[161] Hancocks N. Sudeep nutrition launches Lipoboost liposomal ingredients. (2024). Available online at: https://www.nutraingredients.com/Article/2024/06/19/Sudeep-Nutrition-launches-Lipoboost-liposomal-ingredients/ (Accessed August 26, 2025).

[162] Liposomal supplements market revenue to attain USD 690.80 Mn by 2033. (2025). Available online at: https://www.precedenceresearch.com/press-release/liposomal-supplements-market (Accessed August 26, 2025).

[163] Jupiter neurosciences introduces Nugevia™ MND: A cognitive health supplement leveraging clinically validated neuroscience. (2025). Available online at: https://www.stocktitan.net/news/JUNS/jupiter-neurosciences-introduces-nugevia-tm-mnd-a-cognitive-health-79cm2w84n95a.html (Accessed August 27, 2025).

[164] Bampi GB Backes GT Cansian RL de Matos FE Ansolin IMA Poleto BC . Spray chilling microencapsulation of *Lactobacillus acidophilus* and *Bifidobacterium animalis* subsp. *lactis* and its use in the preparation of savory probiotic cereal bars. Food Bioprocess Technol. (2016) 9:1422–8. doi: 10.1007/s11947-016-1724-z

[165] Uriho A Chen K Zhou F Ma L Chen C Zhang S . Functional breads with encapsulated vitamin C and fish oil: nutritional, technological, and sensory attributes. Antioxidants. (2024) 13:1325. doi: 10.3390/antiox13111325, 39594466 PMC11590905

[166] Agriopoulou S Tarapoulouzi M Varzakas T Jafari SM. Application of encapsulation strategies for probiotics: from individual loading to co-encapsulation. Microorganisms. (2023) 11:2896. doi: 10.3390/microorganisms11122896, 38138040 PMC10745938

[167] Sarika K Jayathilakan K Kumar L Priya E Greeshma S Rajkumar A Omega-3 enriched granola bar: Formulation and evaluation under different storage conditions. Kochi, Kerala, India: Society of Fisheries Technologists. (2019).

[168] Bhutto RA Mahar H Khanal S Wang M Iqbal S Fan Y . Recent trends in co-encapsulation of probiotics with prebiotics and their applications in the food industry. Trends Food Sci Technol. (2025) 156:104829. doi: 10.1016/j.tifs.2024.104829

[169] Kazemeini H Azizian A Adib H. Inhibition of *Listeria monocytogenes* growth in Turkey fillets by alginate edible coating with *Trachyspermum ammi* essential oil nano-emulsion. Int J Food Microbiol. (2021) 344:109104. doi: 10.1016/j.ijfoodmicro.2021.109104, 33676333

[170] Lee J-S Lee HG. Nano-encapsulation of a combination of clove oil and thymol and their application in fresh-cut apples and raw minced beef. Food Control. (2023) 148:109683. doi: 10.1016/j.foodcont.2023.109683

[171] Kolanowski W Weißbrodt J. Sensory quality of dairy products fortified with fish oil. Int Dairy J. (2007) 17:1248–53. doi: 10.1016/j.idairyj.2007.04.005

[172] Yakubu HG Ali O Ilyés I Vigyázó D Bóta B Bazar G . Micro-encapsulated microalgae oil supplementation has no systematic effect on the odor of Vanilla shake-test of an electronic nose. Foods. (2022) 11:3452. doi: 10.3390/foods11213452, 36360065 PMC9654470

[173] Kairam N Kandi S Sharma M. Development of functional bread with flaxseed oil and garlic oil hybrid microcapsules. LWT. (2021) 136:110300. doi: 10.1016/j.lwt.2020.110300

[174] Kouame KJE-P Bora AFM Li X Sun Y Liu L. Novel trends and opportunities for microencapsulation of flaxseed oil in foods: a review. J Funct Foods. (2021) 87:104812. doi: 10.1016/j.jff.2021.104812

[175] Tolve R Bianchi F Lomuscio E Sportiello L Simonato B. Current advantages in the application of microencapsulation in functional bread development. Foods. (2022) 12:96. doi: 10.3390/foods12010096, 36613312 PMC9818201

[176] Lane KE Li W Smith C Derbyshire E. The bioavailability of an omega-3-rich algal oil is improved by nanoemulsion technology using yogurt as a food vehicle. Int J Food Sci Technol. (2014) 49:1264–71. doi: 10.1111/ijfs.12455

[177] Santos DS Morais JAV Vanderlei ÍA Santos AS Azevedo RB Muehlmann LA . Oral delivery of fish oil in oil-in-water nanoemulsion: development, colloidal stability and modulatory effect on in vivo inflammatory induction in mice. Biomed Pharmacother. (2021) 133:110980. doi: 10.1016/j.biopha.2020.110980 33249282

[178] Schneider I Schuchardt JP Meyer H Hahn A. Effect of gastric acid resistant coating of fish oil capsules on intestinal uptake of eicosapentaenoic acid and docosahexaenoic acid. J Funct Foods. (2011) 3:129–33. doi: 10.1016/j.jff.2011.03.001

[179] Haq M Chun B-S. Microencapsulation of omega-3 polyunsaturated fatty acids and astaxanthin-rich salmon oil using particles from gas saturated solutions (PGSS) process. LWT. (2018) 92:523–30. doi: 10.1016/j.lwt.2018.03.009

[180] Ojha KS Perussello CA García CÁ Kerry JP Pando D Tiwari BK. Ultrasonic-assisted incorporation of nano-encapsulated omega-3 fatty acids to enhance the fatty acid profile of pork meat. Meat Sci. (2017) 132:99–106. doi: 10.1016/j.meatsci.2017.04.260, 28558948

[181] Hădărugă NG Chirilă CA Szakal RN Gălan IM Simandi MD Bujancă GS . FTIR–PCA approach on raw and thermally processed chicken lipids stabilized by nano-encapsulation in β-cyclodextrin. Foods. (2022) 11:3632. doi: 10.3390/foods11223632, 36429225 PMC9689604

[182] Raatz SK Johnson LK Bukowski MR. Enhanced bioavailability of EPA from emulsified fish oil preparations versus capsular triacylglycerol. Lipids. (2016) 51:643–51. doi: 10.1007/s11745-015-4100-2, 26688435

[183] McClements DJ. Advances in edible nanoemulsions: digestion, bioavailability, and potential toxicity. Prog Lipid Res. (2021) 81:101081. doi: 10.1016/j.plipres.2020.101081 33373615

[184] Maki KC Dicklin MR. Strategies to improve bioavailability of omega-3 fatty acids from ethyl ester concentrates. Curr Opin Clin Nutr Metab Care. (2019) 22:116–23. doi: 10.1097/MCO.0000000000000537, 30550388

[185] Singh AK Pal P Pandey B Goksen G Sahoo UK Lorenzo JM . Development of “smart foods” for health by nanoencapsulation: novel technologies and challenges. Food Chem. (2023) 20:100910. doi: 10.1016/j.fochx.2023.100910, 38144773 PMC10740092

[186] Augustin MA Sanguansri L. Challenges and solutions to incorporation of nutraceuticals in foods. Annu Rev Food Sci Technol. (2015) 6:463–77. doi: 10.1146/annurev-food-022814-015507, 25422878

[187] Buya AB Mahlangu P Witika BA. From lab to industrial development of lipid nanocarriers using quality by design approach. Int J Pharm. (2024) 8:100266. doi: 10.1016/j.ijpx.2024.100266, 39050378 PMC11268122

[188] Rahim MA Zahran HA Jaffar HM Ambreen S Ramadan MF Al-Asmari F . Liposomal encapsulation in food systems: a review of formulation, processing, and applications. Food Sci Nutr. (2025) 13:e70587. doi: 10.1002/fsn3.70587, 40766785 PMC12321603

[189] Viegas C Patrício AB Prata JM Nadhman A Chintamaneni PK Fonte P. Solid lipid nanoparticles vs. nanostructured lipid carriers: a comparative review. Pharmaceutics. (2023) 15. doi: 10.3390/pharmaceutics15061593, 37376042 PMC10305282

[190] McClements DJ. Recent advances in the production and application of nano-enabled bioactive food ingredients. Curr Opin Food Sci. (2020) 33:85–90. doi: 10.1016/j.cofs.2020.02.004

[191] López KL Ravasio A González-Aramundiz JV Zacconi FC. Solid lipid nanoparticles (SLN) and nanostructured lipid carriers (NLC) prepared by microwave and ultrasound-assisted synthesis: promising green strategies for the nanoworld. Pharmaceutics. (2023) 15:1333. doi: 10.3390/pharmaceutics15051333, 37242575 PMC10221859

[192] Islam F Saeed F Afzaal M Hussain M Ikram A Khalid MA. Food grade nanoemulsions: promising delivery systems for functional ingredients. J Food Sci Technol. (2023) 60:1461–71. doi: 10.1007/s13197-022-05387-3, 37033316 PMC10076486

[193] Wang Y Ai C Wang H Chen C Teng H Xiao J . Emulsion and its application in the food field: an update review. eFood. (2023) 4:e102. doi: 10.1002/efd2.102

[194] Yang J Shen M Luo Y Wu T Chen X Wang Y . Advanced applications of chitosan-based hydrogels: from biosensors to intelligent food packaging system. Trends Food Sci Technol. (2021) 110:822–32. doi: 10.1016/j.tifs.2021.02.032

[195] Reza Mozafari M Johnson C Hatziantoniou S Demetzos C. Nanoliposomes and their applications in food nanotechnology. J Liposome Res. (2008) 18:309–27. doi: 10.1080/08982100802465941, 18951288

[196] Acosta E. Bioavailability of nanoparticles in nutrient and nutraceutical delivery. Curr Opin Colloid Interface Sci. (2009) 14:3–15. doi: 10.1016/j.cocis.2008.01.002

[197] Charreau H Cavallo E Foresti ML. Patents involving nanocellulose: analysis of their evolution since 2010. Carbohydr Polym. (2020) 237:116039. doi: 10.1016/j.carbpol.2020.116039, 32241405

[198] Oberdörster G Stone V Donaldson K. Toxicology of nanoparticles: a historical perspective. Nanotoxicology. (2007) 1:2–25. doi: 10.1080/17435390701314761

[199] Kabanov AV. Polymer genomics: an insight into pharmacology and toxicology of nanomedicines. Adv Drug Deliv Rev. (2006) 58:1597–621. doi: 10.1016/j.addr.2006.09.019, 17126450 PMC1853357

[200] Qi J Zhuang J Lu Y Dong X Zhao W Wu W. In vivo fate of lipid-based nanoparticles. Drug Discov Today. (2017) 22:166–72. doi: 10.1016/j.drudis.2016.09.024, 27713035

[201] Zhou H McClements DJ. Recent advances in the gastrointestinal fate of organic and inorganic nanoparticles in foods. Nano. (2022) 12:1099. doi: 10.3390/nano12071099, 35407216 PMC9000219

[202] des Rieux A Fievez V Garinot M Schneider Y-J Préat V. Nanoparticles as potential oral delivery systems of proteins and vaccines: a mechanistic approach. J Control Release. (2006) 116:1–27. doi: 10.1016/j.jconrel.2006.08.013, 17050027

[203] Powell JJ Faria N Thomas-McKay E Pele LC. Origin and fate of dietary nanoparticles and microparticles in the gastrointestinal tract. J Autoimmun. (2010) 34:J226–33. doi: 10.1016/j.jaut.2009.11.006, 20096538

[204] Mahato DK Mishra AK Kumar P. Nanoencapsulation for Agri-food applications and associated health and environmental concerns. Front Nutr. (2021) 8:663229. doi: 10.3389/fnut.2021.663229, 33898505 PMC8060450

[205] Heringa M Peters R Bleys R Van Der Lee M Tromp P Van Kesteren P . Detection of titanium particles in human liver and spleen and possible health implications. Part Fibre Toxicol. (2018) 15:15. doi: 10.1186/s12989-018-0251-7 29642936 PMC5896156

[206] Bettini S Boutet-Robinet E Cartier C Coméra C Gaultier E Dupuy J . Food-grade TiO2 impairs intestinal and systemic immune homeostasis, initiates preneoplastic lesions and promotes aberrant crypt development in the rat colon. Sci Rep. (2017) 7:40373. doi: 10.1038/srep40373, 28106049 PMC5247795

[207] Yang YX Song ZM Cheng B Xiang K Chen XX Liu JH . Evaluation of the toxicity of food additive silica nanoparticles on gastrointestinal cells. J Appl Toxicol. (2014) 34:424–35. doi: 10.1002/jat.2962, 24302550

[208] McClements DJ Xiao H. Is nano safe in foods? Establishing the factors impacting the gastrointestinal fate and toxicity of organic and inorganic food-grade nanoparticles. Npj Sci Food. (2017) 1:6. doi: 10.1038/s41538-017-0005-1, 31304248 PMC6548419

[209] Sohal IS O’Fallon KS Gaines P Demokritou P Bello D. Ingested engineered nanomaterials: state of science in nanotoxicity testing and future research needs. Part Fibre Toxicol. (2018) 15:29. doi: 10.1186/s12989-018-0265-1, 29970114 PMC6029122

[210] Biswas R Alam M Sarkar A Haque MI Hasan MM Hoque M. Application of nanotechnology in food: processing, preservation, packaging and safety assessment. Heliyon. (2022) 8:e11795. doi: 10.1016/j.heliyon.2022.e11795, 36444247 PMC9699984

[211] Dobrovolskaia MA McNeil SE. Immunological properties of engineered nanomaterials. Nat Nanotechnol. (2007) 2:469–78. doi: 10.1038/nnano.2007.223, 18654343

[212] Fadeel B Farcal L Hardy B Vázquez-Campos S Hristozov D Marcomini A . Advanced tools for the safety assessment of nanomaterials. Nat Nanotechnol. (2018) 13:537–43. doi: 10.1038/s41565-018-0185-0, 29980781

[213] Saraiva C Praça C Ferreira R Santos T Ferreira L Bernardino L. Nanoparticle-mediated brain drug delivery: overcoming blood–brain barrier to treat neurodegenerative diseases. J Control Release. (2016) 235:34–47. doi: 10.1016/j.jconrel.2016.05.044, 27208862

[214] Bouwmeester H Dekkers S Noordam MY Hagens WI Bulder AS De Heer C . Review of health safety aspects of nanotechnologies in food production. Regul Toxicol Pharmacol. (2009) 53:52–62. doi: 10.1016/j.yrtph.2008.10.008 19027049

[215] Jain A Ranjan S Dasgupta N Ramalingam C. Nanomaterials in food and agriculture: an overview on their safety concerns and regulatory issues. Crit Rev Food Sci Nutr. (2018) 58:297–317. doi: 10.1080/10408398.2016.1160363, 27052385

[216] Higashisaka K Yoshioka Y Tsutsumi Y. Applications and safety of nanomaterials used in the food industry. Food Saf. (2015) 3:39–47. doi: 10.14252/foodsafetyfscj.2015005

[217] Chau C-F Wu S-H Yen G-C. The development of regulations for food nanotechnology. Trends Food Sci Technol. (2007) 18:269–80. doi: 10.1016/j.tifs.2007.01.007

[218] Committee ES More S Bampidis V Benford D Bragard C Halldorsson T . Guidance on risk assessment of nanomaterials to be applied in the food and feed chain: human and animal health. EFSA J. (2021) 19:e06768. doi: 10.2903/j.efsa.2021.6768, 34377190 PMC8331059

[219] Oomen AG Bleeker EA Bos PM Van Broekhuizen F Gottardo S Groenewold M . Grouping and read-across approaches for risk assessment of nanomaterials. Int J Environ Res Public Health. (2015) 12:13415–34. doi: 10.3390/ijerph121013415, 26516872 PMC4627040

[220] Committee ES More S Bampidis V Benford D Bragard C Halldorsson T . Guidance on technical requirements for regulated food and feed product applications to establish the presence of small particles including nanoparticles. EFSA J. (2021) 19:e06769. doi: 10.2903/j.efsa.2021.6769, 34377191 PMC8331058

[221] Amenta V Aschberger K Arena M Bouwmeester H Moniz FB Brandhoff P . Regulatory aspects of nanotechnology in the Agri/feed/food sector in EU and non-EU countries. Regul Toxicol Pharmacol. (2015) 73:463–76. doi: 10.1016/j.yrtph.2015.06.016, 26169479

[222] Coles D Frewer LJ. Nanotechnology applied to European food production–a review of ethical and regulatory issues. Trends Food Sci Technol. (2013) 34:32–43. doi: 10.1016/j.tifs.2013.08.006

[223] Duvall MN Knight K. FDA regulation of nanotechnology. Washington, DC, USA: Beveridge and Diamond (2012).

[224] Guidance D. Guidance for industry considering whether an FDA-regulated product involves the application of nanotechnology. Biotechnol Law Rep. (2011) 30:613–6. doi: 10.1089/blr.2011.9814, 41340826

[225] Sadrieh N Espandiari P. Nanotechnology and the FDA: what are the scientific and regulatory considerations for products containing nanomaterials. Nanotech L Bus. (2006) 3:339.

[226] Sandoval B. Perspectives on FDA'S regulation of nanotechnology: emerging challenges and potential solutions. Compr Rev Food Sci Food Saf. (2009) 8:375–93. doi: 10.1111/j.1541-4337.2009.00088.x

[227] Liu L. Asia–Pacific nanotechnology: Research, development, and commercialization. The Nano–Micro Interface: Bridging the Micro and Nano worlds. Weinheim: Wiley. (2004) 35–48.

[228] Liu L. Overview on nanotechnology R&D and commercialization in the Asia Pacific region. The Nano-Micro Interface: Bridging the Micro and Nano worlds. Weinheim: Wiley. (2015) 37–54.

[229] Hunt G Mehta M. Nanotechnology: risk, ethics and law. London: Routledge (Taylor & Francis Group). (2013).

[230] Chuah AS Leong AD Cummings CL Ho SS. Label it or ban it? Public perceptions of nano-food labels and propositions for banning nano-food applications. J Nanopart Res. (2018) 20:36. doi: 10.1007/s11051-018-4126-5

[231] Sodano V Gorgitano MT Verneau F Vitale CD. Consumer acceptance of food nanotechnology in Italy. Br Food J. (2016) 118:714–33. doi: 10.1108/BFJ-06-2015-0226

[232] Yue C Zhao S Cummings C Kuzma J. Investigating factors influencing consumer willingness to buy GM food and nano-food. J Nanopart Res. (2015) 17:283. doi: 10.1007/s11051-015-3084-4

[233] Zhou G Hu W. Public acceptance of and willingness-to-pay for nanofoods in the US. Food Control. (2018) 89:219–26. doi: 10.1016/j.foodcont.2018.02.004

[234] Peters RJ Bouwmeester H Gottardo S Amenta V Arena M Brandhoff P . Nanomaterials for products and application in agriculture, feed and food. Trends Food Sci Technol. (2016) 54:155–64. doi: 10.1016/j.tifs.2016.06.008

[235] Bouwmeester H Brandhoff P Marvin HJ Weigel S Peters RJ. State of the safety assessment and current use of nanomaterials in food and food production. Trends Food Sci Technol. (2014) 40:200–10. doi: 10.1016/j.tifs.2014.08.009

[236] Hu B Huang Q-r. Biopolymer based nano-delivery systems for enhancing bioavailability of nutraceuticals. Chin J Polym Sci. (2013) 31:1190–203. doi: 10.1007/s10118-013-1331-7

[237] Muthukrishnan L. Nanonutraceuticals—challenges and novel nano-based carriers for effective delivery and enhanced bioavailability. Food Bioprocess Technol. (2022) 15:2155–84. doi: 10.1007/s11947-022-02807-2

[238] Dima C Assadpour E Dima S Jafari SM. Nutraceutical nanodelivery; an insight into the bioaccessibility/bioavailability of different bioactive compounds loaded within nanocarriers. Crit Rev Food Sci Nutr. (2021) 61:3031–65. doi: 10.1080/10408398.2020.1792409, 32691612

[239] Yang L-Y Li C-Q Zhang Y-L Ma M-W Cheng W Zhang G-J. Emerging drug delivery vectors: engineering of plant-derived nanovesicles and their applications in biomedicine. Int J Nanomedicine. (2024) 19:2591–610. doi: 10.2147/IJN.S454794, 38505167 PMC10949304

[240] Wang B Zhuang X Deng Z-B Jiang H Mu J Wang Q . Targeted drug delivery to intestinal macrophages by bioactive nanovesicles released from grapefruit. Mol Ther. (2014) 22:522–34. doi: 10.1038/mt.2013.190, 23939022 PMC3944329

[241] Zhuang X Teng Y Samykutty A Mu J Deng Z Zhang L . Grapefruit-derived nanovectors delivering therapeutic miR17 through an intranasal route inhibit brain tumor progression. Mol Ther. (2016) 24:96–105. doi: 10.1038/mt.2015.188, 26444082 PMC4754550

[242] Filipe V Hawe A Jiskoot W. Critical evaluation of nanoparticle tracking analysis (NTA) by NanoSight for the measurement of nanoparticles and protein aggregates. Pharm Res. (2010) 27:796–810. doi: 10.1007/s11095-010-0073-2, 20204471 PMC2852530

[243] Paredes AJ Asensio CM Llabot JM Allemandi DA Palma SD Nanoencapsulation in the food industry: Manufacture, applications and characterization. Bucharest: AMG Transcend Association. (2016).

[244] Rostamabadi H Falsafi SR Jafari SM. Transmission electron microscopy (TEM) of nanoencapsulated food ingredients In: Characterization of nanoencapsulated food ingredients. Cambridge, MA (USA): Elsevier. (2020). 53–82.

[245] Khosravi-Darani K Pardakhty A Honarpisheh H Rao VM Mozafari MR. The role of high-resolution imaging in the evaluation of nanosystems for bioactive encapsulation and targeted nanotherapy. Micron. (2007) 38:804–18. doi: 10.1016/j.micron.2007.06.009, 17669661 PMC7126426

[246] Farooqi MA Kang C-U Choi KH. Organ-on-chip: advancing nutraceutical testing for improved health outcomes. ACS Omega. (2023) 8:31632–47. doi: 10.1021/acsomega.3c03155, 37692213 PMC10483668

[247] Malekjani N Jafari SM. Release modeling of nanoencapsulated food ingredients by empirical and semiempirical models In: Release and bioavailability of nanoencapsulated food ingredients. Cambridge, MA (USA): Elsevier. (2020). 211–46.

[248] Rahbari S Tavakolipour H Kalbasi-Ashtari A. Application of electro-spraying technique and mathematical modelling for nanoencapsulation of curcumin. Heliyon. (2024) 10:e25680. doi: 10.1016/j.heliyon.2024.e25680, 38390193 PMC10881552

[249] Malekjani N Jafari SM. Release modeling of nanoencapsulated food ingredients by mechanistic models In: Release and bioavailability of nanoencapsulated food ingredients. Cambridge, MA (USA): Elsevier. (2020). 247–71.

[250] Djuris J Vidovic B Ibric S. Release modeling of nanoencapsulated food ingredients by artificial intelligence algorithms In: Release and bioavailability of nanoencapsulated food ingredients. Cambridge, MA (USA): Elsevier. (2020). 311–47.

[251] Xu S Basem A Al-Asadi HA Chaturvedi R Daminova G Fouad Y . Employing deep learning for predicting the thermal properties of water and nano-encapsulated phase change material. Int J Low-Carbon Technol. (2024) 19:1453–9. doi: 10.1093/ijlct/ctae098

[252] Abdelsalam SI Alsedais N Aly AM. Revolutionizing bioconvection: artificial intelligence-powered nano-encapsulation with oxytactic microorganisms. Eng Appl Artif Intell. (2024) 137:109128. doi: 10.1016/j.engappai.2024.109128

[253] Wang Y Brahmia A Shahbaz A Sahramaneshi H Alkhalifah T Yang J. A novel machine learning model for innovative microencapsulation techniques and applications in advanced materials, textiles, and food industries. Renew Sust Energ Rev. (2025) 224:116082. doi: 10.1016/j.rser.2025.116082

[254] Campagnolo L Lacconi V Filippi J Martinelli E. Twenty years of in vitro nanotoxicology: how AI could make the difference. Front Toxicol. (2024) 6:1470439. doi: 10.3389/ftox.2024.1470439, 39376973 PMC11457712

[255] Wang Y Lu CD Chen W Wang Q Jiang H. Digital twin enabled personalized nutrition. Precis Nutr. (2023) 2:e00030. doi: 10.1097/PN9.0000000000000030

[256] Joshi S Shamanna P Dharmalingam M Vadavi A Keshavamurthy A Shah L . Digital twin-enabled personalized nutrition improves metabolic dysfunction-associated fatty liver disease in type 2 diabetes: results of a 1-year randomized controlled study. Endocr Pract. (2023) 29:960–70. doi: 10.1016/j.eprac.2023.08.016, 37778441

[257] Li Y Pei Y Shan Z Jiang Y Cui SW He Z . A pH-sensitive W/O/W emulsion-bound carboxymethyl chitosan-alginate hydrogel bead system through the Maillard reaction for probiotics intestine-targeted delivery. Food Hydrocoll. (2024) 153:109956. doi: 10.1016/j.foodhyd.2024.109956

[258] Zheng M Jia H Qiu M Yang X Zhang X Zhao Q . Recent advances in stimuli-responsive oral delivery systems for controlled release of probiotics: a review. Food Res Int. (2025) 220:117132. doi: 10.1016/j.foodres.2025.117132, 41074371

[259] Zhao S Zhao Y Yang X Zhao T. Recent research advances on oral colon-specific delivery system of nature bioactive components: a review. Food Res Int. (2023) 173:113403. doi: 10.1016/j.foodres.2023.113403, 37803751

[260] Thwaites PA Yao CK Halmos EP Muir JG Burgell RE Berean KJ . Current status and future directions of ingestible electronic devices in gastroenterology. Aliment Pharmacol Ther. (2024) 59:459–74. doi: 10.1111/apt.17844, 38168738 PMC10952964

[261] Zheng Z Zhu R Peng I Xu Z Jiang Y. Wearable and implantable biosensors: mechanisms and applications for closed-loop therapeutic systems. J Mater Chem B. (2024) 12:8577–604. doi: 10.1039/D4TB00782D, 39138981

[262] Ghanim R Kaushik A Park J Abramson A. Communication protocols integrating wearables, ingestibles, and implantables for closed-loop therapies. Device. (2023) 1. doi: 10.1016/j.device.2023.100092

[263] Ramezani G Stiharu I van de Ven TG Nerguizian V. Advancements in hybrid cellulose-based films: innovations and applications in 2D nano-delivery systems. J Funct Biomater. (2024) 15:93. doi: 10.3390/jfb15040093 38667550 PMC11051498

[264] Chang L Wang Y-C Ershad F Yang R Yu C Fan Y. Wearable devices for single-cell sensing and transfection. Trends Biotechnol. (2019) 37:1175–88. doi: 10.1016/j.tibtech.2019.04.001, 31072609 PMC9245325

[265] Tripathy S Verma DK Gupta AK Srivastav PP Patel AR González MLC . Nanoencapsulation of biofunctional components as a burgeoning nanotechnology-based approach for functional food development: a review. Biocatal Agric Biotechnol. (2023) 53:102890. doi: 10.1016/j.bcab.2023.102890

[266] Tripathy S Srivastav PP. Encapsulation of *Centella asiatica* leaf extract in liposome: study on structural stability, degradation kinetics and fate of bioactive compounds during storage. Food Chem Adv. (2023) 2:100202. doi: 10.1016/j.focha.2023.100202

[267] Linares-Castaneda A Franco-Hernandez MO Gómez y Gómez YM Corzo-Rios LJ. Physical properties of zein-alginate-glycerol edible films and their application in the preservation of chili peppers (*Capsicum annuum* L.). Food Sci Biotechnol. (2024) 33:889–902. doi: 10.1007/s10068-023-01393-z, 38371689 PMC10866812

[268] Jahangiri F Mohanty AK Misra M. Sustainable biodegradable coatings for food packaging: challenges and opportunities. Green Chem. (2024) 26:4934–74. doi: 10.1039/D3GC02647G

[269] Niu B Zhan L Shao P Xiang N Sun P Chen H . Electrospinning of zein-ethyl cellulose hybrid nanofibers with improved water resistance for food preservation. Int J Biol Macromol. (2020) 142:592–9. doi: 10.1016/j.ijbiomac.2019.09.134, 31739036

[270] Fallahasghari EZ Stubbe PR Chronakis IS Mendes AC. Ethyl cellulose-core, OSA starch-shell electrosprayed microcapsules enhance the oxidative stability of loaded fish oil. Nano. (2024) 14:510. doi: 10.3390/nano14060510, 38535657 PMC10974531

[271] Ahuja A Rastogi VK. PFAS free, food-grade, water and grease-resistant coating based on crosslinked shellac for molded pulp products. Prog Org Coat. (2024) 196:108734. doi: 10.1016/j.porgcoat.2024.108734

[272] Jahangiri F Mohanty A Pal AK Clemmer R Gregori S Misra M. Wax coatings for paper packaging applications: study of the coating effect on surface, mechanical, and barrier properties. ACS Environ Au. (2024) 5:165–82. doi: 10.1021/acsenvironau.4c00055, 40125279 PMC11926751

[273] Miranda M Sun X Marín A Dos Santos LC Plotto A Bai J . Nano-and micro-sized carnauba wax emulsions-based coatings incorporated with ginger essential oil and hydroxypropyl methylcellulose on papaya: preservation of quality and delay of post-harvest fruit decay. Food Chem. (2022) 13:100249. doi: 10.1016/j.fochx.2022.100249 PMC9040031 35499002

[274] Junior PCG Bertagnolli C da Silva CAM Braga MB. Microencapsulation of a flaxseed and avocado oil blend: influence of octenyl succinic anhydride (OSA)-modified starch and rice and pea proteins on powder characterization and oxidative stability. PRO. (2024) 12:2230. doi: 10.3390/pr12102230

[275] Ayar-Sumer EN Nyambe C Hashim MA Altin-Yavuzarslan G El-Messery TM Ozçelik B. Optimizing encapsulation of black carrot extract using complex coacervation technique: maximizing the bioaccessibility and release kinetics in different food matrixes. Lwt. (2024) 198:115995. doi: 10.1016/j.lwt.2024.115995

[276] Xiao Z Xia J Zhao Q Niu Y Zhao D. Maltodextrin as wall material for microcapsules: a review. Carbohydr Polym. (2022) 298:120113. doi: 10.1016/j.carbpol.2022.120113, 36241287

[277] Šeregelj V Ćetković G Čanadanović-Brunet J Šaponjac VT Vulić J Lević S . Encapsulation of carrot waste extract by freeze and spray drying techniques: an optimization study. LWT. (2021) 138:110696. doi: 10.1016/j.lwt.2020.110696

[278] Gutiérrez-Pacheco MM Ortega-Ramírez LA Silva-Espinoza BA Cruz-Valenzuela MR González-Aguilar GA Lizardi-Mendoza J . Individual and combined coatings of chitosan and carnauba wax with oregano essential oil to avoid water loss and microbial decay of fresh cucumber. Coatings. (2020) 10:614. doi: 10.3390/coatings10070614

[279] Chit C-S Olawuyi IF Park JJ Lee WY. Effect of composite chitosan/sodium alginate gel coatings on the quality of fresh-cut purple-flesh sweet potato. Gels. (2022) 8:747. doi: 10.3390/gels8110747, 36421569 PMC9689777

[280] Zhou X Guo N Zhang F Zhuo K Zhu G. Improving stability and bioavailability of ACNs based on Gellan gum-whey protein isolate nanocomplexes. Food Chem. (2024) 24:102050. doi: 10.1016/j.fochx.2024.102050, 39703377 PMC11656087

[281] Zhang X Liu D Jin TZ Chen W He Q Zou Z . Preparation and characterization of gellan gum-chitosan polyelectrolyte complex films with the incorporation of thyme essential oil nanoemulsion. Food Hydrocoll. (2021) 114:106570. doi: 10.1016/j.foodhyd.2020.106570

